# Supplementary figures and images for: Effects of executive function training on balance and auditory-cognitive dual-task performance in adults with and without hearing loss
Source: PLoS One. 2026 Apr 29;21(4):e0331276. doi: 10.1371/journal.pone.0331276 (PMC13127936; doi:10.1371/journal.pone.0331276)

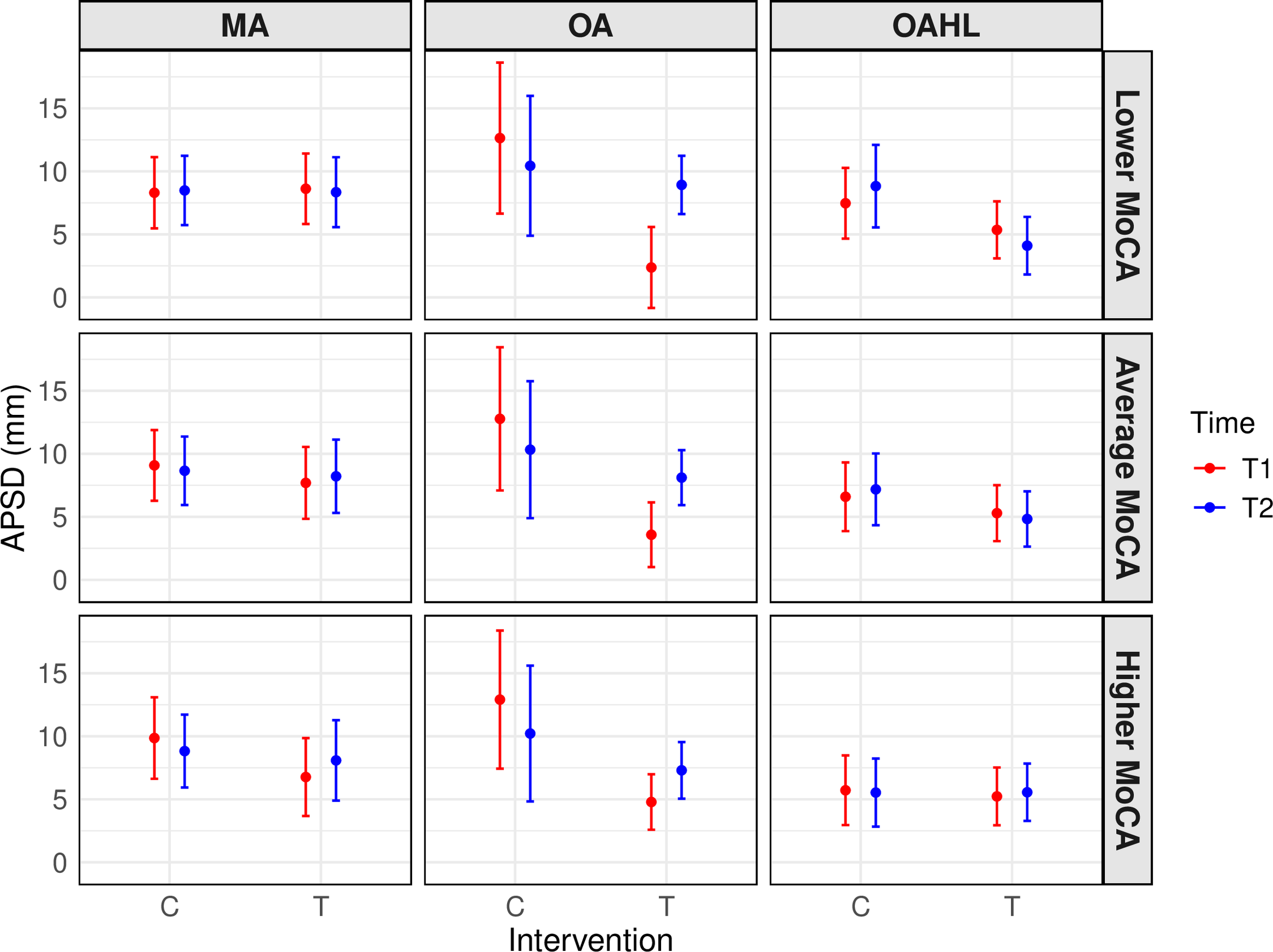

Supplement: S2 Fig — Estimated marginal means of Centre of Pressure Path Length Anterior-Posterior Standard Deviation (COP APSD in mm; higher scores represent worse performance) from T1 to T2 for those in both the executive function cognitive training (T) and control (C) conditions. Group and Montreal Cognitive Assessment (MoCA) tertiles are represented by the columns and rows respectively. Asterisks indicate statistically significant training-related improvements. Errors bars represent the 95% confidence intervals. (TIF) [file pone.0331276.s001.tif]

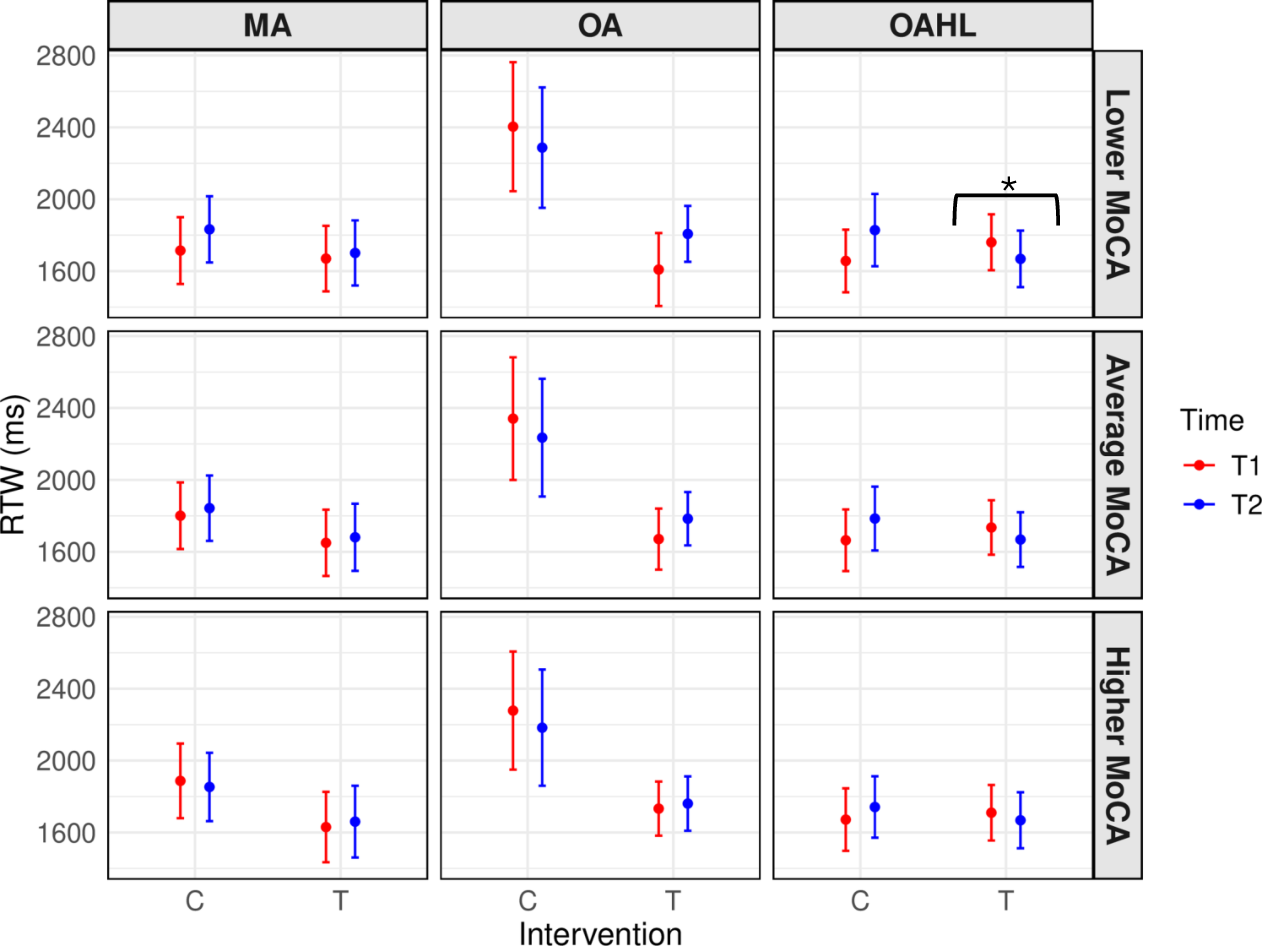

Supplement: S3 Fig — Estimated marginal means of auditory 2-back Reaction Time Weighted (RTW in ms, higher scores represent worse performance) from T1 to T2 for both the Executive Function training (T) and control (C) conditions. Group and Montreal Cognitive Assessment (MoCA) tertiles are represented by the columns and rows, respectively. Asterisks indicate statistically significant training-related improvements. Errors bars represent the 95% confidence intervals around model-estimated marginal means, which may extend beyond observed data ranges (i.e., max of 2000 ms) due to statistical estimation. (TIF) [file pone.0331276.s002.tif]
