# Supplementary material for: Effects of executive function training on balance and auditory-cognitive dual-task performance in adults with and without hearing loss
Source: PLoS One. 2026 Apr 29;21(4):e0331276. doi: 10.1371/journal.pone.0331276 (PMC13127936; doi:10.1371/journal.pone.0331276)
Supplement: S1 File — (DOCX) [file pone.0331276.s003.docx]

# **Supporting Information**

## **S1. Baseline Measures**

To assess our secondary objective (that is, whether sensory, cognitive and motor functions as evaluated by standardized assessments of functioning predict training-related outcomes on experimental auditory 2-back and balance outcomes), the following assessments were completed by all participants at both T1, a subset of which were completed again at T2. Those tests that were not completed at T2 are delineated with an * and the tests used to determine eligibility are underlined below.

*Hearing*

**Pure-Tone Audiometry***: Hearing acuity was evaluated using the SHOEBOX^TM^ Audiometer [1]. Tones at frequencies of 250 Hz, 500 kHz, 1 kHz, 2 kHz, 4 kHz, and 8 kHz were delivered through headphones to each ear at varying intensity levels. Participants were asked via tablet (iPad; Apple Inc.) to indicate whether a tone was detected. For each ear, the pure-tone average (PTA) across the 500 Hz, 1 kHz, 2 kHz, and 4 kHz tones were calculated. Higher threshold values on the test indicated worse performance.

**Canadian Digit Triplet Test (CDTT):** To evaluate participant’s threshold for identifying speech in noise, the CDTT was administered [2]. Higher speech reception threshold (SRT in dB SNR) values on this test correspond to worse performance.

**Hearing Handicap Inventory for the Elderly (HHIE)*:** Self-reported challenges with everyday hearing were evaluated using the HHIE online questionnaire (maximum score = 40) where higher scores denote increased hearing-related difficulty [3].

**Listening Self-Efficacy Questionnaire (LSEQ):** Self-perceived listening ability in a range of everyday auditory scenarios were evaluated using the LSEQ questionnaire online (maximum score = 18), higher scores represent better performance [4].

*Cognition*

**Montreal Cognitive Assessment (MoCA):** The MoCA is used to screen for mild cognitive impairment and assesses various cognitive abilities (i.e., executive function (EF), memory, attention, language, abstraction, visuospatial skills, and orientation). The MoCA is adjusted for years of education (maximum score = 30), higher scores indicate better cognitive performance [5].

**Rey Auditory Verbal Learning Test (RAVLT):** The RAVLT (maximum score = 75) was administered to assess verbal learning and memory retention, with a delayed recall trial (RAVLT Delayed, maximum score = 15) conducted 20–30 minutes later. Alternate test versions were used at T1 and T2 to minimize practice effects. Higher scores reflected stronger performance [6].

**Digit Span Forward, Backward & Letter-Number Sequencing (LNS):** Verbal short-term memory was evaluated using the Digit Span Forward subtest from the Wechsler Adult Intelligence Scale (WAIS-IV; score out of 16), while auditory working memory was assessed via the Digit Span Backward subtest (maximum = 14) and the LNS subtest (maximum = 30) [7]. Since the LNS assessment was only completed for the middle-aged adults with normal hearing (MA; aged 45-60 years), we have reported the mean and standard deviation here (*M* = 18.95, *SD* = 2.57) instead of in the main demographics table (see Table 1 in the paper). In all three subtests, higher scores denote better memory performance.

**Digit Symbol-Coding:** The Digit Symbol-Coding subtest (score out of 133) was used to measure visual-motor processing speed, with higher scores indicating faster and more accurate performance. During the task, numbers ranging from 1 to 9 were each associated with a symbol. Participants were instructed to draw the correct symbol for each number and complete this for as many numbers as possible (total of 133 numbers) in 2 minutes [7].

**Stroop Color-Word Interference:** EF was assessed using the Color-Word Interference Test (maximum score = 19), which evaluated word reading, color naming, inhibitory control, and cognitive flexibility/switching. Scaled scores—based on age and completion time—were used. The inhibitory and switching scores are reported in the demographics table (see Table 1 in paper). Higher scores reflect better EF performance [8].

**Trail Making Test (TMT):** The Trail Making Test (TMT) assessed processing speed and cognitive flexibility through subtests A and B. A difference score (subtest B minus subtest A) was calculated (seconds), with larger values indicating poorer performance due to increased difficulty switching between cognitive tasks. For TMT subtest A, participants saw a page with spatially distributed numbers ranging from 1 to 25 and were instructed to draw a line from one number to the next in consecutive order (e.g., 1 to 2, 2 to 3 and so on until 25). For TMT subtest B, participants saw a page with spatially distributed numbers ranging from 1 to 13 and letters ranging from A to L and were instructed to draw a line from one number to letter in consecutive order (e.g., 1 to A, A to 2, 2 to B and so on until 13) [9].

**Frequency of Forgetting Questionnaire (FFQ)**: The FFQ Questionnaire was administered online to evaluate subjective memory performance and the regularity with which participants experience forgetfulness in daily life (maximum score = 10). Higher scores reflect worse performance. Participants self-reported their general frequency of forgetting on a scale from 1 (major problems) to 7 (no problems). Then participants self-reported how often different stimuli present a problem for them (e.g., frequency of forgetting names, faces, previous sentence they read by) on a scale from 1(always) to 7 (never). Lastly, participants indicated their long-term memory abilities (e.g., memory for things which occurred 1-5 years ago) on a scale from 1 (very bad) to 7 (very good) [10].

*Vision*

**Early Treatment Diabetic Retinopathy Study** **(ETDRS)*:** To ensure participants had normal or corrected-to-normal vision, they were tested using the ETDRS chart. Higher values indicate worse vision. Participants were instructed to read an eye chart with their left eye and then their right eye. A score of 0.0 logarithm of the Minimum Angle of Resolution (logMAR) is considered normal vision [11].

**Pelli-Robson Contrast Sensitivity (PRCS)**: To measure visual contrast sensitivity, participants completed the PRCS test; higher scores reflect better visual performance. Participants were presented with an eye chart that had letters which faded from left to right and top to bottom. Specifically, the top line had high contrast letters; that is, black letters on a white background which turned grey as the letters move down the chart. Participants were instructed to read the chart with their left eye, right eye and then both eyes. A score of 1.8 logarithmic contrast sensitivity (logCS) and higher is considered normal contrast sensitivity [12].

*Mobility*

**Mini-BESTest:** Motor functioning was evaluated using the Mini-BESTest (maximum score = 28), a shortened version of the Balance Evaluation Systems Test. This assessment provides quantitative measures of postural control across four key balance systems: anticipatory movements (e.g., sit-to-stand transitions), reactive responses to instability (e.g., regaining balance after leaning beyond the base of support), sensory orientation (e.g., maintaining stability with visual or surface challenges), and dynamic gait (e.g., walking while turning the head, varying speed, or navigating obstacles). Higher scores indicate better performance [13].

**Activities-specific Balance Confidence (ABC)**: Participants' self-reported confidence in their ability to maintain balance during everyday tasks was measured using the ABC Scale online (maximum score = 100). Higher scores indicate better performance [14].

*Other*

**Health History Questionnaire (HHQ)*:** An online HHQ was administered online to collect information on participants' personal health and demographic background to better characterize participants and to screen for eligibility for the study.

## **S2. Balance Performance**

Here we report the full statistics of the results that were not reported in the paper and are being reported separately below in order to maintain a succinct paper. Specifically, we included all the statistics that pertain to an improvement in training in the paper and all other full statistics below with elaboration.

First, in order to address our primary objective of investigating the effects of EF training across time and groups on standing balance performance, a linear mixed-effect model (LMM) was conducted with the primary balance outcome of interest (i.e., Centre of Pressure Anterior-Posterior Standard Deviation; COP APSD; mm) as the dependent variable, where higher values indicate worse performance. First, we found a significant four-way Intervention*Time*Age*PTA interaction on COP APSD performance, *F*(1,425.11) = 5.0022, *p* = .026, *η_p_^2^* = .01). Post-hoc tests revealed that for the EF training condition at younger ages and average levels of hearing loss, balance performance at T1 (time 1) was significantly lower/better than T2 (time 2, *t* = -3.401, *p*<.001). Second, for the EF training condition at average ages and average levels of hearing loss, balance performance at T1 was significantly lower/better than T2 (*t* = -3.368, *p*<.001). Third, for the EF training condition at older ages and average levels of hearing loss, balance performance at T1 was significantly lower/better than T2 (*t* = -2.701, *p* = 0.007). Fourth, for the EF training condition at younger ages and higher levels of hearing loss, balance performance at T1 was significantly lower/better than T2 (*t* = -3.189, *p* = .002). Next, for the EF training condition at averages ages and higher levels of hearing loss, balance performance at T1 was significantly lower/better than T2 (*t* = -3.691, *p*<.001). Lastly, for the EF training condition at older ages and higher levels of hearing loss, balance performance at T1 was significantly lower/better compared to T2 (*t* = -4.055, *p*<.001). Overall, for all ages (younger, average or older) who had the worst hearing loss (i.e., average-higher levels of hearing loss), there was no positive effects of EF training (i.e., balance performance got worse).

Second, we found a significant four-way Intervention*Time*Group*MoCA interaction on COP APSD performance, *F*(2,426.06) = 8.8496, *p*<.001, *η_p_^2^* = .04. Post-hoc tests revealed that for the older adults with normal hearing (OA) EF training condition, for those with lower MoCA scores, balance performance at T1 was significantly lower/better than T2 (*t* = -5.179, *p*<.001). Secondly, for the OA EF training condition, for those with average MoCA scores, balance performance at T1 was significantly lower/better than T2 (*t* = -4.621, *p*<.001). Thirdly, for the OA EF training condition for those with higher MoCA scores, balance performance at T1 was significantly lower/better than T2 (*t* = -2.772, *p*= .006). Likewise, for the older adults with hearing loss who used hearing aids (OAHL) control condition, for those with lower MoCA scores, balance performance at T1 was significantly lower/better compared to T2 (*t* = -1.968, *p* = .050; see S2 Fig below). Overall, for those in the OA EF training condition, regardless of their MoCA level, there were no positive effects of EF training on balance performance. In the OAHL control condition, for those with lower MoCA scores, there were no T2 improvements in balance performance.

## **S3. Auditory 2-back Performance**

First, in order to address our primary objective of investigating the effects of EF training across time and groups on auditory 2-back performance, an LMM was conducted with the primary auditory 2-back reaction time weighted (RTW, ms) score outcome of interest, as the dependent variable, for which higher values indicate worse performance. First, we found a significant four-way Intervention*Time*Age*PTA interaction effect on auditory 2-back RTW performance, *F*(1,418.43) = 4.6998, *p* = .031, *η_p_^2^* = .01. Post-hoc tests revealed that, for those in the control condition who were at average ages and had lower PTAs, at T1 RTW was significantly lower/better than at T2 (*t* = -2.525, *p* = .012). Further, for those in the control condition who were older and had lower PTAs, at T1 RTW was significantly lower/better than at T2 (*t* = -2.779, *p* = .006). Overall, those assigned to the no-treatment control condition generally worsened over time in auditory 2-back performance.

Second, we found a significant four-way Intervention*Time*Group*MoCA interaction effect on auditory 2-back RTW performance, *F*(2,425.46) = 8.7397, *p*<.001, *η_p_^2^* = .04. Post-hoc tests revealed that for those in the OA EF training condition who had lower MoCA scores, at T1 RTW was significantly lower/better than at T2 (*t* = -2.803, *p* = .005). Further, for those in the OA EF training condition who had average MoCA scores, at T1 RTW was significantly lower/better than at T2 (*t* = -1.999, *p* = .046). Likewise, for those in the OAHL control condition who had lower MoCA scores, at T1 RTW was significantly lower/better than at T2 (*t* = -2.507, *p* = .013). Also, for those in the OAHL control condition who had average MoCA scores, at T1 RTW was significantly lower/better than at T2 (*t* = -2.269, *p* = .024; see S3 Fig below). Overall, those in the OA EF training condition with lower, or average MoCA levels demonstrated worse auditory 2-back performance at T2 compared to T1. Those assigned to the no-treatment control condition generally worsened over time on auditory 2-back performance.

**References**

1. Bastianelli M, Mark AE, McAfee A, Schramm D, Lefrançois R, Bromwich M. Adult validation of a self-administered tablet audiometer. Journal of Otolaryngology-Head & Neck Surgery. 2019 Jan;48(1):59.
2. Giguère C, Lagacé J, Ellaham NN, Pichora-Fuller MK, Goy H, Bégin C, et al. Development of the Canadian digit triplet test in English and French. The Journal of the Acoustical Society of America. 2020 Mar 1;147(3):EL252-8.
3. Weinstein BE, Ventry IM. Audiometric correlates of the hearing handicap inventory for the elderly. Journal of Speech and Hearing Disorders. 1983 Nov;48(4):379-84.
4. Smith SL, Kathleen Pichora-Fuller M, Watts KL, La More C. Development of the listening self-efficacy questionnaire (LSEQ). International Journal of Audiology. 2011 Jun 1;50(6):417-25.
5. Nasreddine ZS, Phillips NA, Bédirian V, Charbonneau S, Whitehead V, Collin I, et al. The Montreal Cognitive Assessment, MoCA: a brief screening tool for mild cognitive impairment. Journal of the American Geriatrics Society. 2005 Apr;53(4):695-9.
6. Lezak MD. Neuropsychological assessment. Oxford University Press, USA; 2004.
7. Wechsler D. Wechsler Adult Intelligence Scale WAIS - IV Canadian. San Antonio, TX: Pearson; 2008.
8. Delis DC, Kaplan E, Kramer JH. Delis-Kaplan executive function system. Assessment. 2001 Jan 1.
9. Reitan RM. The Halstead-Reitan neuropsychological test battery: therapy and clinical interpretation. (No Title). 1985.
10. Zelinski EM. Far transfer in cognitive training of older adults. Restorative neurology and neuroscience. 2009 Oct;27(5):455-71.
11. Ferris III FL, Kassoff A, Bresnick GH, Bailey I. New visual acuity charts for clinical research. American journal of ophthalmology. 1982 Jul 1;94(1):91-6.
12. Pelli DG, Robson JG, Wilkins AJ. The design of a new letter chart for measuring contrast sensitivity. Clin Vis Sci. 1988;2(3):187–99.
13. Franchignoni F, Horak F, Godi M, Nardone A, Giordano A. Using psychometric techniques to improve the Balance Evaluation System’s Test: the mini-BESTest. Journal of rehabilitation medicine: official journal of the UEMS European Board of Physical and Rehabilitation Medicine. 2010 Apr;42(4):323.
14. Powell LE, Myers AM. The activities-specific balance confidence (ABC) scale. The journals of Gerontology Series A: Biological sciences and Medical sciences. 1995 Jan 1;50(1):M28-34.
