# Supplementary material for: Effects of executive function training on balance and auditory-cognitive dual-task performance in adults with and without hearing loss
Source: PLoS One. 2026 Apr 29;21(4):e0331276. doi: 10.1371/journal.pone.0331276 (PMC13127936; doi:10.1371/journal.pone.0331276)
Supplement: S3 File — CAPCR submission form. (PDF) [file pone.0331276.s005.pdf]

CAPCR Submission Form

CAPCR-ID: 19-5857.0

Study Title: Walking while listening - the impact of hearing impairment on mobility in older adults

Study Nickname: Walking while listening

Initial: New Study

Date Submitted: 15 Apr 2020

PI's Name: Jennifer Campos
PI's Email: jennifer.campos@uhn.ca
PI's Phone #: 416-597-3422 Ext 7958
PI's Location: Toronto Rehabilitation Institute, University Centre, 550 University Avenue, Room 12-173, Toronto, Ontario, Canada, M5G 2A2

Study Contacts:
N/A

Prepared by:

Niroshica Mohanathas, niroshica.mohanathas@uhn.ca
Katherine Bak, katherine.bak@uhn.ca, 416-597-3422 x7803

Submission Documents:
Consent Form - Appendix B. Screening, Consent...
Recruitment Materials (advertisement, etc.) - Appendix C. Recruitment Materials (Post)
Recruitment Materials (advertisement, etc.) - Appendix D: Recruitment Materials (Fly)
Qualitative Study Instruments (questionnaires, etc.) - Appendix E. Testing Assessments
Protocol - Appendix A. Protocol_NM
Miscellaneous Document - Appendix B. Funding
Qualitative Study Instruments (questionnaires, etc.) - Appendix U. Subjective Cognitive
Qualitative Study Instruments (questionnaires, etc.) - Appendix V. Executive Function Training
Consent Form --Tracked - Appendix G. Informed Consent Form_Tracke
Consent Form - Appendix G. Informed Consent Form_Clean

Reviewed by:
Department/Division Head
RQI Training
CTA (Clinical Trial Agreements)
TRI KITE - StreetLab
REB (Research Ethics Board)
Data Registry Sub-Committee

Walking while listening

CAPCR ID: 19-5857.0

STUDY BASIC INFORMATION

1. **Full Study Title:**

   Walking while listening - the impact of hearing impairment on mobility in older adults

2. **Study Nickname:**

   Walking while listening

3. **Is there a protocol number or identifier for this study?**

   Yes

   If yes, complete the following question:

   3a. **Specify the protocol number/identifier.**

   19-5857.0

4. **Expected start date of the study at this institution:**

   1-Oct-2019

5. **Expected end date of the study at this institution:**

   30-Mar-2023

6. **Department/Division/Program Head:**

   Milos Popovic (milos.popovic@uhn.ca)

7. **Site(s) where this study will take place**

   TRI

STUDY OVERVIEW

1. **ABSTRACT (Suitable for a public access or lay audience):**

Navigating the environment involves successfully integrating your visual, auditory and vestibular system. However, this becomes more challenging for older adults who experience comorbid deficits in age-related hearing loss (ARHL) and mobility and are at an increased risk for falls. The "cognitive compensation" hypothesis states that hearing loss causes increased "cognitive load" because listening effort is increased. This load could take important cognitive resources away from behaviours that support safe mobility. Importantly, the interdependence of cognitive, sensory, and motor declines can lead to physical deconditioning, reduced out-of-home activity, and social withdrawal, even in individuals with sub- clinical levels of hearing loss.

2. **Background of the study: provide a summary of findings from previous studies (pre-clinical and clinical) that lead to the conduct of this study.**

Age Effect: Although existing strategies such as hearing aids can amplify noise in quiet environments for individuals with hearing loss, these devices are limited when detecting signals in noise in more complex environments: background noise, multi-talker situations or auditory-mobility activities (e.g., balancing-while-listening when crossing the street at an intersection). However, these more complex dual-tasking situations are more realistic representations of the daily situations faced by older adults. Hearing loss is also the top, mid-life potentially modifiable risk factor for dementia. This supports the idea that preventing sensory declines in middle-aged adults can be beneficial for a variety of reasons.

Training Effect: Experimental studies provide convergent evidence of the involvement of higher-level cognitive processing in auditory task performance (e.g., speech understanding in noise, auditory working memory, language comprehension) when sensory demands are high or concurrent task demands are added. Under dual-task conditions in which a concurrent task competes for cognitive capacity, older adults exhibit greater dual-task costs in listening than younger adults, suggesting that listening becomes more cognitively effortful with aging. This recruitment of cognitive capacity to support auditory processing, even when hearing loss is sub-clinical, leaves less capacity for higher-level functions such as memory encoding, language comprehension, or other attention-demanding tasks such as walking. Given the known age-related declines in higher-level cognitive abilities such as executive function (switching,

working memory, inhibition), the potential for effective cognitive compensation could benefit from targeted Executive Function Training. Executive Function Training will especially be beneficial to those experiencing severe sensory impairments.

3. **What is the rationale for this study?**

The current study will use a state-of-the-art virtual reality simulator called Streetlab, given the complexity of sensory- motor integration required for function in the community. Both older adults and middle aged-adults will perform a battery of sensory, motor and cognitive tasks. Our main hypothesis is that all participants who undergo Executive Function Training (but not the waitlist control group) will show improved balancing-while-listening task performance, with the greatest training-related gains in the ARHL group. The proposed work is novel in its inclusion of normal-hearing middle-aged adults and older hearing aid users in addition to older adults with normal hearing. This will enable us to address the timely clinical and policy-relevant issues of early prevent on and the added value of Executive Function Training in combination with hearing aid use (see Appendix B-Funding).

4. **What are the study hypotheses or research questions?**

Our main hypothesis is that all participants who undergo cognitive training (and not the waitlist control) will show improved walking and listening task performance, with the greatest training-related gains in the ARHL group.

5. **Describe the primary objectives and briefly describe how they will be measured. Describe the secondary objectives and how they will be measured (if applicable).**

A) Primary Outcomes: To see the effect of cognitive training on complex auditory and motor functioning under simulated real-word conditions.

B) Secondary Outcomes: To better understand the broader impact of cognitive training, we will also assess perceptual and cognitive functioning, and subjective measures of everyday functioning before and after training.

There will be three sessions: pre-assessment, the cognitive training intervention followed by post-assessment. The pre- and post-assessment sessions include psychophysical and pencil-and-paper tasks of sensory, physical, and cognitive function. The cognitive intervention will involve training on a computer or tablet in the comfort of participant homes.

6. **What is the significance of the study (i.e. the overall anticipated public and/or scientific benefit)?**

Over 30% of individuals over age 65 experience one or more falls per year, leading to significant health care costs. The cost of fall-related injuries in Canada is estimated at $2 billion annually. Age-related mobility decline and fall risk is associated with reduced cognitive capacity, and with reduced activity of the frontal brain regions that subserve executive function and link to motor regions. The risk of falls is tripled for older adults with a hearing impairment (defined as ≥ 25 dB HL pure-tone average thresholds) compared to those with normal hearing. Yet many older adults with clinically "normal" hearing or untreated mild hearing loss experience difficulty in challenging listening environments (in background noise or multi-talker situations) or in auditory-motor activities (e.g., listening while walking). A recent meta-analysis indicates that although hearing aids and auditory training can improve simple auditory task performance, Executive Function Training is needed to improve the more complex listening activities associated with everyday functioning.

MULTI-SITE STUDY

1. **What is the lead/coordinating site?**

UHN is the lead institution

2. **Indicate whether the study will be conducted at any of the TAHSN sites.**

None

3. **Who developed the protocol for this study?**

UHN PI

MULTI-SITE STUDY SITES

1. **Please specify the non-UHN sites.**

   Concordia University, PERFORM Centre

STUDY PERSONNEL - PRINCIPAL INVESTIGATOR

1. **Department/Division**

   Research

2. **Program**

   N/A

3. **Site/Organization**

   TRI

   If TRI, complete the following question:

   3a. **The proposed research is associated with the PI's work as part of the following TRI research team(s):**

   Home, Community & Institutional Environments Team

4. **Does the PI require access to EPIC?**

   No

STUDY PERSONNEL

The following information is to be supplied for each member of the study team, except the PI.

Note: All personnel involved in the conduct of the study at UHN should be listed in this section, including for example, co-investigators, data abstractors and study managers. Omission of study team members from the Study Personnel list may cause a delay in the review of your submission.

**1. First and Last Name**	Niroshica Mohanathas
**Email**	niro.mohanathas@mail.utoronto.ca
**2. Department/Division:**	Research
**3. Program:**	Psychology
**4. Site/Organization:**	TRI
**5. Role(s) in study:**	Study Coordinator; Study Manager; Research Student
**6. Appointment expiration date (for time-limited role such as student or research fellow):**	1-Oct-2024
**7. Does this person require access to EPR (Electronic Patient Record) in order to perform this role?**	No

CONSENT

1. **Describe the consent process.**

   The consent form will be sent electronically via email to all participants after the prescreening interview (to see if participants are eligible for the study) ensuring to use the UHN file portal with the appropriate level of security for sending the document. If participants have any questions they will be encouraged to ask these questions via email or phone. Participants will sign the form when they arrive to their first study visit in the presence of the study coordinator. There will be two copies of the consent form, one for the participant to take home upon completion of the experiment and one for the research student to keep as a record.

2. **Who will obtain consent?**

   The experimenters: Niroshica Mohanathas, or other research assistants

3. **Is there a relationship between the participants and the person obtaining consent?**

   No

4. **Is there a relationship between the participants and the investigator?**

   No

5. **How much time will be given to participants to review the information before being asked to give consent?**

   As much time as they need.

6. **Does this study involve any participants who may lack the ability to provide informed consent for the duration of the study?**

   No

7. **Does this study involve any participants who may initially lack the ability to provide informed consent, but may gain or regain it at some point during the study?**

   No

8. **Does the study involve any participants who may have communication difficulties?**

   No

CONTRACTS

1. **The following information is to be supplied for each party external to UHN that will be entering into an agreement (contract) with the institution.**
   Note: A contract/agreement may be required for many reasons, for example: if the study involves multiple sites; if there is any transfer of data, materials, etc.; if there is a transfer of funds; if a medical device is being developed, built, or distributed in connection with a party outside UHN. This section is included in your CAPCR form because you have indicated that your study requires a contract or agreement. Do not answer N/A to questions in this section.

**1.1. Has the agreement been submitted (outside of CAPCR) to CTA or TDC for review and signing?**	Yes	Yes
**1.2. Has the agreement been signed by a UHN signing authority?**	Yes	Yes
**1.3. Name(s) and contact**		

**information of the other party to the agreement**		
**1.3a. Party's full name**	Karen Li	Rachel Downey
**1.3b. Party's institution**	Concordia	Concordia
**1.3c. Party's full address**	L-PY 131-4<br>Psychology Building,<br>7141 Sherbrooke W.	L-PY 131-4<br>Psychology Building,<br>7141 Sherbrooke W.
**1.3d. Party's email address**	Karen.Li@concordia.ca	downeyr01@gmail.com
**1.4. Role of the other party:**	Collaborating institution	Collaborating institution
**1.5. Are biospecimens being transferred to or from this party?**	No	No
**1.6. Is data being transferred to or from this party?**	Transfer both to and from this party	Transfer both to and from this party
**1.6a. Specify what kinds of data will be transferred**	Anonymous; results/data from experimental measures	Anonymous; Results/data from experimental measures.
**1.7. Are funds being transferred to or from this party?**	Transfer FROM this party to UHN	Transfer FROM this party to UHN

2. **Are there any other agreements or grants related to this study?**

No

DATA COLLECTION AND USE

1. **Detailed description of data gathering processes and procedures**

40 middle-aged adults with normal hearing (40 - 60 years), 40 older adults with normal hearing (60+ years) and 40 age-matched older adults (60+ years) who are experienced hearing aid users (≥ 6 months regular use, audiometric threshold ≥ 40 dB HL @ 2 kHz) will be recruited by phone and email and will be screened over the phone and in-person for exclusion and inclusion criteria. This session has 3 parts.

The first testing session will include a battery of sensory, motor and cognitive tests. Upon completion of this session, if you are interested and eligible you will be invited for another assessment. The assessment will take place at Toronto Rehab's "virtual reality simulator. The virtual environment used in this study will resemble several city blocks surrounding Toronto Rehab. You will be asked to perform multiple trials of a) walking down the street at a comfortable pace on a treadmill, b) listening to numbers and sentences and identifying numbers and words and, c) simultaneously walking while performing the listening task. Upon completion of this, you will be asked to complete a series of questionnaires and computerized tasks online in the comfort of your own home for approximately 30-minute sessions, 3 times per week, for 12 weeks. After the 12 weeks you will be requested to return to Toronto Rehab to complete the sensory, motor and cognitive tests and the Virtual Reality walking-while-listening task again, which will be the third session (for additional details refer to Appendix A).

2. **Describe the methods that will be used to analyze study data. Please provide references to the applicable page(s) of the protocol.**

We will conduct preliminary treatment group comparisons on all background variables to ensure adequate randomization across training conditions. The pre-training experimental data will be analyzed with Age Group (middle, older, ARHL) and Cognitive Load (single- vs. dual-task) as factors to confirm previously reported age and dual-task effects (e.g., auditory task accuracy, gait velocity).

Our main hypothesis is that all participants who undergo Executive Function Training (and not the active control group) will show improved walking and listening task performance, with the greatest training-related gains in the ARHL group. We will conduct mixed-factorial ANOVAs to assess the effects of Group (middle- aged, older, ARHL), Treatment (training vs. control) and Time (pre- vs. post-training) on the primary outcome variables from the StreetLab site (spatial listening accuracy, 2-back accuracy, kinematics). Should there be any group confounds detected in the preliminary analysis of the secondary outcomes (e.g., pure tone audiometry, CDTT, etc.), we will include covariates in these analyses. For the secondary outcomes, we will conduct confirmatory factor analyses to determine if the outcome measures (e.g., pure audiometry, CDTT, etc.) can be combined to form compound variables (e.g., hearing) to address the problem of multiple outcomes and spurious findings. If warranted, we will then create compound scores (averaged z-scores) and subject these to similar ANOVAs, with anticipated improvements to cognitive, motor and auditory functioning in the Executive Function Training groups. Finally, for only those participants who undergo the Executive Function Training, regression analyses will be conducted to evaluate the relationship between training gains on the Executive Function Training (mean RT slopes) and change scores on the outcome variables (refer to Appendix A for more details).

3. **Are any interim data analyses planned?**

Yes

If yes, complete the following question

3a. **Describe the interim analysis plans.**

Descriptive (e.g., mean, SD) of our dependent measures will be obtained after collecting participants data. This will be done to ensure proper data capture and integrity before collecting the full data set.

4. **Indicate how study participants will be identified in study data (e.g. study number, initials).**

Pre-screening: Initially when calling, participant's names will be linked to the pre-screening questionnaire using an ID number. The excel sheet linking participant's names and their corresponding ID number will be password protected and stored on the TRI network drive.

Recruitment: Once the participants have successfully met eligibility, their pre-screening form, consent form, health history questionnaire and all other identifying information will be de-identified and placed in a separate binder behind closed doors that require security access, separate from all testing documents.

Data Transcription and Presentation: All participants will be de-identified by using just their ID number to identify them (no identifiable information like name or email will be linked to their data from the study). All public dissemination of results will not identify any individuals and the project data will be stored for 10 years.

5. **Will any information collected through this study be linked with any other databases external to UHN? (e.g. other health care institutions, health registries, Statistics Canada)?**

No

FUNDING

1. **The following information is to be supplied for each funding source:**

**1.1. Name of company, granting agency, internal funding source, or other funding source:**	CIHR
**1.2. Type of funding source:**	Government Funding Agency - Canada (e.g. Canadian Institutes for Health Research)

**1.3. What is the status of funding from this source?**	Obtained

2. **If any requested funding is not received, will you be able to proceed with the study?**

   No

3. **If all requested funding is received, will it be sufficient to cover all study costs?**

   Yes

4. **Is this study receiving any Tri Council funding (CIHR, NSERC, SSHRC)?**

   Yes

5. **Is this study receiving any NCIC funding?**

   No

6. **Is this study receiving any US federal funds?**

   No

7. **Is this research supported by the United States federal government?**

   No

8. **Will the study require a grant account, FC (Functional Centre) or IO (Internal Order), now or in the future?**

   Yes

   If yes, complete the following question:

   8a. **Will an existing RFS account be used?**

   410012572

FUNDING - EXISTING GRANT ACCOUNT

1. **The following information is to be supplied for each existing grant account for this study:**

**1. Grant account / FC (Functional Centre) / IO (Internal Order) number from existing grant(s):**	410012572

PERSONAL HEALTH INFORMATION

1. **Specify all personal health information required to be collected for the conduct of the study, including study recruitment activities**

   Name;
   Phone/fax number;
   Email/IP address/URL;
   Date of birth(year only);
   Health information (e.g. related to inclusion/exclusion criteria, medications, laboratory results)

2. **Identify sources of personal health information.**

   Database maintained by PI

3. **Explain why this study cannot reasonably be accomplished without using the PHI outlined in your response above.**

   Participant's contact information (e.g., email and phone number) is used to contact the

participant. In order to characterize individuals, age is required to separate participants into three groups: normal-hearing middle-aged adults (40-60 years of age) and older hearing aid users (60 + years of age) in addition to normal- hearing older adults (60 + years of age). The health and demographic information can help us ensure that all participants do indeed meet all inclusion and exclusion criteria, if something was inaccurately reported at the prescreening interview stage.

4. **Will any personal health information will be sent outside of UHN?**

No

5. **What are the risks if PHI collected for the purposes of this study were released to an unauthorized party?**

No Greater Than Minimal Risk: Privacy breached.

6. **If PHI were disclosed to an unauthorized party, what specific procedures, methods or controls would be implemented to minimize the potential harms?**

Any sensitive information will be de-identified. Names will not be attached with sensitive information or asked in sensitive documents. As well, the following will be done; further release of information will be stopped, any information that can be retrieved will be retrieved, the UHN Privacy Office and REB will be notified, and then further actions may be taken according to recommendations from the UHN Privacy Office and REB.

PERSONAL HEALTH INFORMATION - STORAGE

1. **Will the storage of study records conform to the requirements of the 'Storage, Transport & Destruction of Confidential Information' policy?**

Yes

PERSONAL HEALTH INFORMATION - TRANSPORT

1. **Will the transport of study records conform to the requirements of the 'Storage, Transport & Destruction of Confidential Information' policy?**

Yes

PERSONAL HEALTH INFORMATION - DESTRUCTION

1. **Will the destruction of study records conform to the requirements of the 'Storage, Transport & Destruction of Confidential Information' policy?**

Yes

PROSPECTIVE STUDY DESIGN

1. **Type of study**

Lab-based behavioral study

2. **Describe the study design and methodology (provide a standalone synopsis of the study. Include: type of study (pilot, phase I, II, III, IV, RCT, qualitative, etc.), procedures (screening, intervention arm, control arm, Questionnaire, Group Discussion, Interview, etc.), duration and study visits, sub-studies (mandatory, optional, etc.)**

We will collect information on health and demographics, eligible participants will proceed to the in-person assessment sessions.

A) Session 1 (Pre-Training): Core Assessment (1.5):
All participants will undergo assessment pre-training to test the following outcomes (tests also used for screening are underlined): (see Appendix A and J-M)
(1) audition: hearing acuity pure tone audiometry and the Canadian Digit Triplets Test (CDTT)
(2) balance and mobility: Mini-BEST
(3) vision: ETDRS eye chart, DVA and Pelli-Robson Contrast Sensitivity Test

(4) cognition: global cognitive status, processing speed, auditory working memory, task switching, response inhibition, auditory verbal memory, Montreal Cognitive Assessment (MoCA), Stroop, Wechsler Adult Intelligence Scale (WAIS)-IV Digit Symbol Substitution and Letter-Number-Sequencing, Trail Making Test A&B, and Rey Auditory Verbal Learign test (RAVLT).

B) Session 1: (Pre-training) StreetLab Auditory-motor Dual-task Assessment (1.5 h):
We will use StreetLab to simulate everyday walking (street crossing) while listening. Participants will be presented with 12 randomly ordered single digits and instructed to indicate (yes/no) if they hear a match between the current item and the one presented two items back (n-back 2). Next, participants will perform the multi-talker spatial listening task, in which a visual cue indicates which of three simultaneously presented but spatially distributed sentences to report Coordinate Response Measure (CRM).

C) Session 2: At Home Cognitive Training Intervention:
All Executive Function training participants will undergo training over 12 weeks (3x per week, 30 mins/session) on a home computer or tablet and control participants will engage in internet and computer lessons for the 12-week period.

D) Session 3: (Post-training) Core Assessments and Streetlab (1.5 hr)
All participants will repeat section A-B (excluding pure tone audiometry, ETDRS, DVA and Pelli-Robson).

3. **Does this study include control group(s)?**

Yes

If yes, complete the following question

3a. **Indicate the rationale for control group(s).**

If the experimental group improves after training in comparison to the control group, then the control group is used to validate the effectiveness of training. Previous studies have shown that this active control protocol does not lead to improvements in cognition or mobility.

4. **Will a placebo be used?**

No

5. **Does study involve deception or intentional lack of disclosure?**

No

6. **Does this study involve qualitative components?**

Yes

If Yes, complete the following question

6a. **Specify the qualitative components of the study:**

Questionnaires/surveys

7. **Will study participants be subject to restrictions (e.g. lifestyle) during the study?**

No

8. **Describe the circumstances under which a participant may be withdrawn from the study.**

Participants may be withdrawn from the study if they do not meet inclusion or exclusion criteria:

Inclusion:
-Proficient in English (learned before age 5).
-Can ambulate ≥ 10 meters independently.
-Absence of cognitive impairment (MoCA score ≥ 26/30).
-Normal or corrected-to-normal visual acuity (ETDRS).
-Availability of a home computer or tablet with internet connection.
-Age (middle-aged adults: 40-60, older adults: 60+ years of age)

Exclusions:
-Reported major depression, substance abuse or significant psychiatric disorder.
-Uncorrected visual impairment.
-Uncorrected vestibular impairment.
-Parkinson's disease or other neurological disorder or sequelae.
-Clinically significant musculoskeletal disorders, diseases affecting the ear, or damage to the ear (e.g., occupational noise).
-Onset of hearing loss prior to adulthood.

Participants have the ability to withdraw from the study at any time if they wish. Researchers will withdraw a participant from the study if they cannot complete tasks or later on meet an exclusion criteria that was not found at the prescreening interview stage of the project. However, it will be made explicitly clear in the prescreening session and/or when they come into participate through the consent form that there will be no penalties for withdrawing from the study at any time and that all data will be deleted.

PROSPECTIVE STUDY DESIGN - PARTICIPANTS

1. **Total global study enrollment (including UHN and non-UHN)**

   120

2. **Approximate size of eligible population from institution/practice (number, or number/year)**

   0

3. **Total number of participants you wish to recruit at UHN:**

   3a. **Total number of patients you wish to recruit at UHN.**

   0

   3b. **Rate of accrual (patients/month)**

   0

   3c. **Total number of non-patient participants you wish to recruit at UHN.**

   120

4. **Provide a brief summary of sample size justification. Also provide protocol reference (max 5000 characters).**

   We will recruit 3 groups:
   1) 40 middle-aged adults with normal hearing (40- 60 years old)
   2) 40 older adults with normal hearing (60+ years old)
   3) 40 age-matched older adults (60+ years old) who are experienced hearing aid users (≥ 6 months regular use, audiometric threshold ≥ 40 dB HL @ 2
   kHz).

   A convenience sample size of 40 participants in in each group was chosen based on previous studies in the literature. 20 of these participants will be in the EF training group and the other 20 will be in the control group; 10 of whom are male and 10 of whom are female (refer to protocol, figure 1 for details).

5. **Time period for enrolment**

   3-4 months

6. **List the main inclusion and exclusion criteria pertinent to this study (max 10000 characters).**

   Inclusion:
   -Proficient in English (learned before age 5).
   -Can ambulate ≥ 10 meters independently.
   -Absence of cognitive impairment (MoCA score ≥ 26/30).
   -Normal or corrected-to-normal visual acuity (ETDRS).
   -Availability of a home computer or tablet with internet connection.

-Age (middle-aged adults: 45-60, older adults: 65-80 years of age)

Exclusions:
-Reported major depression, substance abuse or significant psychiatric disorder.
-Uncorrected visual impairment.
-Uncorrected vestibular impairment.
-Parkinson's disease or other neurological disorder or sequelae.
-Clinically significant musculoskeletal disorders, diseases affecting the ear, or damage to the ear (e.g., occupational noise).
-Onset of hearing loss prior to adulthood.

7. **Are there any age, ethnicity, language, gender or race-related inclusion or exclusion criteria?**

Yes

If yes, complete the following question

7a. **Provide justification for inclusion/exclusion criteria.**

1) Age: We are interested in the sensory, cognitive and mobility differences between middle aged- adults and older adults with and without hearing loss. Aside from testing older adults, which has been done in the literature before, the novelty of this project is the added middle-aged group, as hearing loss is a preventable risk factor for dementia during mid-life.

2) Language: Inability to speak and understand English is required to complete tasks.

3) Gender: There is a greater prevalence and severity of hearing loss in men compared to women. Specifically, estrogen appears to have a protective effect in pre-menopausal women (e.g., sex effect) and at the same time, hearing impairment is more prevalent in those exposed to high-intensity noise which may occur more frequently in male-dominated jobs such as construction or factory work (e.g., gender effect). This is why each age group will be stratified by sex and gender.

8. **Does this study involve any of the following special populations:**

Healthy volunteers;
Staff;
Older adults, middle aged-adults, hearing loss

PUBLICATION

1. **How will results be communicated to participants?**

Individual debriefing at end of test session

2. **How will results be communicated to other stakeholders?**

Presentation;
Publication

2.1. **How will you publish the results? (Check all that apply.)**

Jointly with co-author(s) from other sites (UHN as a co-lead author with other site)

3. **Has the funding agency or sponsoring company placed any restrictions on publication of findings?**

No

Walking while listening

RECRUITMENT - NON-PATIENTS

1. **What tools will be used to identify potential participants for recruitment into this study?**

   A list of participant's contact information who have agreed to be contacted for future studies located on our network drive and password protected; handing out and posting advertisements upon approval (e.g., posters, flyers at local areas around UHN such as, UofT, Starbucks, Tim Hortons, etc.), web-based recruitment tools [e.g., lab website (mive.ca), the Principal Investigator's (Dr. Jennifer Campos) twitter account (@jlcampos11), the research coordinator's (Niroshica Mohanathas) twitter account (@niro_mohann)].

2. **Who will identify potential study participants?**

   Graduate student (Niroshica Mohanathas) or other research assistants on the project.

3. **Who will make initial contact with potential participants?**

   Graduate student (Niroshica Mohanathas) or other research assistants on the project.

4. **How will contact be made?**

   By phone; By e-mail

REIMBURSEMENT AND COMPENSATION

1. **Will participants be reimbursed for expenses they incur as a result of study participation?**

   No expenses will be incurred

2. **Will participants or substitute decision makers receive compensation for study participation?**

   Yes

   If yes, complete the following question

   2a. **Describe compensation (value, type), including justification.**

   You will be reimbursed as follows for transportation, meals, time, inconvenience: $60 for session 1 pre-training ($30 at the end of the Core Sensory, Cognitive, Motor Assessment and $30 after the Streetlab Assessment), $30 after the Cognitive Training Intervention and $30 after the post-training session ($120 as a total for the entire study). If participants withdraw from the study due to a personal choice or due to the experimenter, they will be paid for the duration of time they completed.

3. **Who will cover reasonable out-of pocket expenses to ensure that immediate medical care is provided if a participant suffers an injury as a result of participation in this study?**

   Institution

RELATED STUDY

These questions refer to a previously approved study at this institution which is directly related to this study (e.g. sub-study, extension, rollover, subsequent to a pilot study).

1. **Who was the Principal Investigator for the previous study?**

   Dr. Jennifer Campos

2. **Specify CAPCR or REB ID number for the previous study.**

   11-033

3. **Specify study title of the previous study. Provide a brief summary of safety and efficacy from that study as it relates to this submission.**

   Title: Listening while Walking.

   Relationship to this submission: The project builds on previous work showing that "listening is

negatively affected by concurrent balance or walking demands, and that ARHL exacerbates dual-task costs in complex listening"; rationale for evaluating cognitive training as a potential intervention to improve functioning is developed with appropriate evidence. The current proposed study is using the same word recognition task in Streetlab following the n-back task, to examine if cognitive training will improve auditory-motor performance.

RISKS, BENEFITS, SAFETY

1. **Potential benefit to study participants**

   There is no direct benefit for study participants.

2. **List the known risks of study intervention(s) in order of approximate rates of occurrence, severity and rates of reversibility (highest to lowest).**

   Overall: Risk is no greater than minimal

   Risks (highest to lowest):
   -Harness In the event of loss of balance
   -Fatigue from having multiple experimental sessions that may be considered long in duration (1.5 hours)
   -Discomfort from standing/sitting
   -Discomfort from eyestrain
   -Evaluation-related discomfort (from the presence of the experimenter or research assistant)

3. **List the risks of any tests, procedures or other protocol-mandated activities that are conducted for research purposes only, including approximate rates of occurrence, severity and reversibility.**

   N/A

4. **Are there any reproductive risks for participants (such as teratogenicity or embryotoxicity of the investigational product, any risk with breastfeeding, or risk to men regarding conception)?**

   No

5. **Is there a safety monitoring plan for this study?**

   Yes

   If yes, complete the following question

   5a. **Provide details of safety monitoring plan.**

   All participants will be monitored during all sessions of the experiment by the graduate student (Niroshica Mohanathas) or research assistant(s).

TRI KITE - STREETLAB

1. **Indicate the resources the study will use from this program or facility.**

   Staff;
   Equipment;
   Facilities;
   Administrative Data;
   Research Data;
   Health Data

A. Study Classification

1.1. Will the study at UHN be using an external ethics review process and/or system (e.g. CTO, OCREB) to obtain REB approval? No
Note: if your study is being reviewed by Veritas, please answer NO

1.2 This submission includes a proposal to: (Check all that apply.)
.

1.2a. **Investigate a current research question** — Yes

1.2b. **Collect and store data and/or biospecimens for future use** — No
This includes creation of a data base or biobank.

1.2c. **Extract and store retrospective data for future use** — No

1.2d. **Conduct quality assurance and quality improvement studies, program evaluation activities, and performance reviews, or testing within normal educational requirements when used exclusively for assessment, management or improvement purposes** — No

2. **Does the study involve any interaction with participants?** — Yes
Answer YES if you are using any data that does not yet exist. For example: interventional studies; questionnaire/survey/interview; prospective (future) collection of biospecimens.
Answer NO if you are using ONLY data or samples that already exist. For example: retrospective chart review; use of previously collected biospecimens.

Does the study involve:

2.1. **Patients receiving care at Princess Margaret Cancer Centre?** — No
Select yes if the intention of the study is to recruit patients with a diagnosis of cancer or the study's objective is to prevent or screen for cancer, even if recruitment/care is occurring at TGH/TWH/TRI.

2.2. **Patients receiving care at any of the Toronto Rehabilitation Institute (TRI) sites?** — No
This includes patients followed up from acute care to Toronto Rehabilitation Institute.

2.3. **Patients receiving care at Toronto General Hospital?** — No

2.4. **Patients receiving care at Toronto Western Hospital?** — No

2.5. **Patients receiving care at any other UHN or non-UHN site?** — No

2.6. **Non-patient participants?** — Yes
For example: Healthy volunteers, students, health care professionals, UHN personnel

2.6a. **Do you wish to use the TRI research volunteer pool to recruit *non-patients* as potential study subjects/participants?** — No
The research volunteer pool is currently closed. You may change the response from YES to NO, or leave unchanged. Do not select YES.

2.7. **Is this a clinical trial as per the World Health Organization (WHO) definition?** — No

2.8. **Does this study involve the use of drugs, natural health products, biologics, genetic therapies, or radioactive drugs?** — No

3.	**Does the study involve access to or use of data that does not yet exist (prospective data)?** ***For example: medical records not yet collected, medical images/reports for scans not yet performed, questionnaires that will be collected as part of the study and databases where the data has not yet been collected.***	Yes
4.	**Does the study involve access to or use of data that already exists (retrospective data)?** ***For example: medical records that have already been collected, medical images/reports for scans that have already been performed, questionnaires that have previously been collected, databases where the data has already been collected.***	No
5.	**Does the study involve use of biospecimens or their derivatives that have not yet been collected (prospective biospecimens)?** ***For example: tissue, blood, body fluids, DNA, RNA, proteins, etc. that will be collected during the study***	No
6.	**Does the study involve use of biospecimens or their derivatives that have already been collected (retrospective biospecimens)?** ***For example: tissue, blood, body fluids, DNA, RNA, proteins, etc. that have previously been collected.***	No
7.	**Are you seeking consent from any individuals for participation in this study?**	Yes
	**From whom are you seeking consent?**	
7.1.	**Patients** ***For example: current or former in-patients or out-patients (UHN or non-UHN)***	No
7.2.	**Non-patients** ***For example: Healthy volunteers, students, health care professionals, UHN personnel***	Yes
	**In what form will consent be obtained? (Select all that apply.)**	
7.3.	**Written consent with signature**	Yes
7.4.	**Other (e.g. verbal, implied)**	No
7.5.	**Do you also plan to enroll any individuals in the study without first obtaining consent?**	No
7.6.	**Will any personal health information be collected, used or disclosed without consent from the individuals to whom the data and/or biospecimens relate?**	No
8.	**Is anyone at UHN, other than the Principal Investigator, involved in the conduct of this study?** ***Include all personnel involved in the study, for example, staff listed on the protocol; students working for the PI; co-investigators; data abstractor; study manager, etc.***	Yes
8a.	**Is this research primarily conducted by personnel who are on a time-limited work term?** ***For example, students, fellows, residents, visiting collaborators***	Yes

9.	**Does this study involve research of a seasonal nature?**	No
10.	**Is the focus of the research primarily in the field of rehabilitation?**	Yes
11.	**Will any research be conducted using any staff, resources, equipment, or facilities, or any administrative, research, or health data, of the Toronto Rehabilitation Institute, including the KITE Centre for Rehabilitation Research?**	Yes
12.	**Is this study directly related to a previously approved study at this institution (e.g. sub-study, extension, rollover, subsequent to a pilot study)?**	Yes
13.	**Is the research being conducted *exclusively* in a country other than Canada?**	No
14.	**Is the UHN PI the sponsor of the study (investigator-initiated study)?**	Yes
15.	**Does the PI have an appointment in the UHN Oncology Program?**	No

B. Risks and Safety

1.	**Has this study undergone prior scientific/scholarly review?**	Yes
2.	**Has this study or a related study undergone review by a Research Ethics Board in Canada?**	Yes
3.	**Will the study have a Data and Safety Monitoring Board (DSMB)?**	No
4.	**Will the study have a Steering Committee?**	No
5.	**Will the research involve the use of biological agents on UHN premises as outlined in the UHN Research Biosafety Manual?**	No
6.	**Does this study involve radioactive material or radiation treatment devices? (PET scans, Bone scans, PDR, HDR, Accelerator, Brachytherapy seeds, Gammaknife)**	No
7.	**Will any member of the immediate study team be handling or potentially exposed to hazardous materials (other than radioactive materials, TDG Class 7)?**	No
8.	**Will any member of the study team be involved in activities with dangerous goods (other than radioactive materials, TDG Class 7) as defined by the Transportation of Dangerous Goods Regulations (TDGR)?** ***Includes packaging, transporting, offering for shipment, receiving dangerous goods such as biohazardous materials, hazardous chemicals, dry ice.***	No
9.	**Does anyone involved in the conduct of this study have a Conflict of Interest (actual, apparent, perceived, or potential)?**	No
10.	**Will the proposed research involve the use of cannabis at UHN?** ***Cannabis means a cannabis plant that belongs to the genus Cannabis and anything referred to in Schedule 1 of the Cannabis Act but does not include***	No

anything referred to in [Schedule 2 of the Cannabis Act](). For more information contact cannabis@uhnresearch.ca .

C. Resources and Services

1.	**Does the study require any services from the Laboratory Medicine Program (LMP) at UHN?** ***Answer 'Yes' if any of the following is involved in the study: Anatomic Pathology; Cytopathology; Molecular/Genetic; Biochemistry; Hematopathology; Flow Cytometry; Hematology; Blood Transfusion Services (Blood Bank); Histocompatibility (HLA); Specimen Management; Microbiology; Coagulation.***	No
2.	**Does this study involve genetic research?**	No
3.	**Does the study use any medical imaging?**	No
4.	**Does the study impact ECG?**	No
5.	**Does the study impact ECHO?**	No
6.	**Is this a multicentre study (i.e. are non-UHN sites involved)?** ***Answer YES if any research-related activities are occurring at non-UHN sites (such as participant recruitment, enrolment, consenting, data collection, data analysis, etc.)***	Yes
7.	**Will any party external to UHN be entering into an agreement or contract with UHN in connection with this research?**	Yes
8.	**Does the study involve any transfer of data, materials, human resources, etc. to/from any party outside UHN, or use of third-party software?** ***"Materials" includes investigational products, biological specimens, etc.***	Yes
9.	**Is there any transfer of funds to or from UHN for any purpose related to this study?**	Yes
	**9a. Will an existing grant account be used?**	Yes
10.	**Does the study involve any of the Health Professions, as an investigator, study participant, or carrying out study activities?** ***Answer YES if any of the following is involved in the study: Anesthesia Assistants; Chiropody; Clinical Nutrition; Kinesiology; Nursing; Occupational Therapy; Physiotherapy; Psychology; Radiation Therapists; Respiratory Therapy; Social Work; Speech Language Pathology; Spiritual Care; Therapeutic Recreation***	No
11.	**Do you require new space for this study?**	No
12.	**Does the study use any medical devices or equipment?** ***For example, hardware, software, mobile apps, accessories, disposables***	No
13.	**Does the study involve any reusable devices or equipment that require sterilization, high-level disinfection, or reprocessing of equipment or medical**	No

devices?

14.	**Does the study involve the use of the Operating Room?**	No
15.	**Will the study impact the provision of anesthesia services, or increase the risk associated with any of these services?** ***Anesthesia services include: general anesthesia, sedation, perioperative pain control***	No
16.	**Does the study use any device that sends, receives, or stores data?**	Yes

G. TRI Facilities

Which of the following TRI programs and/or facilities will be used in connection with this study?

1.	**Brain** ***Including Acquired Brain Injury (ABI), Stroke, LIFEspan, Chronic Pain, Multiple Sclerosis (MS), Complex Injury Outpatient Rehabilitation (CIOR), and Neurology***	No
2.	**Spinal Cord**	No
3.	**Cardiac Rehab**	No
4.	**Geriatric Rehab**	No
5.	**Geriatric Psychiatry**	No
6.	**Musculoskeletal**	No
7.	**Ambulatory Care**	No
8.	**South 5 - Complex Continuing Care**	No
9.	**South 4 - Complex Continuing Care**	No
10.	**South 3 - Complex Continuing Care including Dialysis**	No
11.	**North 3 - Low Tolerance Long Duration (LTLD)**	No
12.	**North 5 - Palliative Care**	No
13.	**North 5 - Special Care**	No
14.	**Speciality Clinics and Augmentative and Alternative Communication (AAC) Clinic**	No
15.	**University Center (UC) Dental Services**	No
16.	**Bickle Center (BC) Dental Services**	No
17.	**KITE HomeLab**	No
18.	**KITE CareLab**	No

**19. KITE FallsLab**	No
**20. KITE ClimateLab**	No
**21. KITE WinterLab**	No
**22. KITE StreetLab**	Yes
**23. KITE StairLab**	No
**24. KITE DriverLab**	No
**25. KITE PerceptionLab**	No
**26. Swallowing Lab**	No
**27. SleepdB Lab**	No
**28. Sleep Clinic**	No
**29. TACOSLab**	No
**30. Movement EvaluationLab**	No
**31. Mobility InnovationsCentre**	No
**32. RELLab**	No
**33. Rumsey CardiacLab**	No
**34. Rumsey NeuroLab**	No
**35. Rocket Family Upper Extremity Clinic-UC**	No
**36. Rocket Family Upper Extremity Clinic-LC**	No
**37. SCI MobilityLab**	No
**38. Other TRI program/facilities**<br>***Additional information must be supplied in the TRI NOTES section***	No

CAPCR Submission Form

CAPCR-ID: 19-5857.1

Study Title: Walking while listening - the impact of hearing impairment on mobility in older adults

Study Nickname: Walking while listening

Research Ethics Renewal

Date Submitted: 17 Aug 2021

PI's Name: Jennifer Campos
PI's Email: jennifer.campos@uhn.ca
PI's Phone #: 416-597-3422 Ext 7958
PI's Location: Toronto Rehabilitation Institute, University Centre, 550 University Avenue, Room 12-173, Toronto, Ontario, Canada, M5G 2A2

Study Contacts:
N/A

Prepared by:
Niroshica Mohanathas, niroshica.mohanathas@uhn.ca

Submission Documents:
N/A

Reviewed by:
REB (Research Ethics Board)

Expiry Date: 28-Sep-2025

Full Board Meeting requested: No

STUDY SUMMARY - PROSPECTIVE STUDY

1. **Provide a brief summary of the progress of the study to date (e.g. recruitment issues, preliminary findings, qualitative study enrolment). For multicentre studies: specify total sample size and number of participants enrolled at other centers. If the study is conducted in phases, e.g. dose escalation and dose expansion parts; Phase I and Phase II; indicate which Part/Phase is currently enrolling participants.**

   Progress: recruitment is delayed due to the pandemic.
   Total global study enrollment (including UHN and non-UHN): 120.

   We will recruit 3 groups:
   1) 40 middle-aged adults with normal hearing (40- 60 years old)
   2) 40 older adults with normal hearing (60+ years old)
   3) 40 age-matched older adults (60+ years old) who are experienced hearing aid users (≥ 6 months regular use, audiometric threshold ≥ 40 dB HL @ 2 kHz).
   A convenience sample size of 40 participants in in each group was chosen based on previous studies in the literature. 20 of these participants will be in the EF training group and the other 20 will be in the control group; 10 of whom are male and 10 of whom are female (refer to protocol, figure 1 for details).

2. **Is there any new information in the literature or from other recent studies that would change the rationale or risk/benefit ratio for this study (e.g. changes in standard of care, new information about side effects, approval of another drug for this indication, etc.)?**

   No

3. **Have any participants been withdrawn from the study intervention prematurely, or withdrawn consent?**

   No

4. **Have there been any participant complaints or feedback about the study?**

   No

5. **Since the last renewal, have all reportable events (e.g. unexpected deaths or serious adverse events related to study participation, etc.) been reviewed in a timely fashion by the PI?**

   There have not been any reportable events since the last renewal

6. **Since the last renewal, have all reportable events been reported to the REB?**

   There have not been any reportable events since the last renewal

7. **Is the study being conducted in accordance with the documents currently approved by the UHN Research Ethics Board?**

   Yes

8. **Since the last renewal, has there been any change in the Conflict of Interest information for study personnel involved in this study, that has not been reported to the REB?**

   No

9. **Are you requesting a "Full Board" REB meeting?**

   No

10. Is this study receiving any US federal funds? No

11. Is this research supported by the United States federal government? No

STUDY PARTICIPANTS - PROSPECTIVE STUDY (RENEWAL)

1.	**Total number of participants approved by the UHN REB to be enrolled at UHN**	120
2.	**Number of charts reviewed to determine eligibility for enrollment**	0
3.	**Number of participants consented to date at UHN**	0
3a.	**Number of patient participants**	0
3b.	**Number of non-patient participants**	0
4.	**Number of Participants:**	
4.1.	**Consented but did not meet inclusion criteria**	0
4.2.	**Consented but have not yet started intervention/data collection**	0
4.3.	**Receiving study intervention (e.g. study drug, questionnaires, tests, or procedures done for study purposes)**	0
4.4.	**In post-intervention follow-up**	0
4.5.	**Have completed the study and no further contact for study purposes is planned**	0
4.6.	**Have withdrawn their consent**	0
4.7.	**Have been withdrawn prematurely by the PI**	0

STUDY PARTICIPANTS - MULTICENTRE STUDY

1. **For multicentre studies, indicate the number of participants enrolled at other centres.**

   To be determine- data collection has not occurred due to the pandemic. However, this external institution is aiming to collect the same sample size as our UHN site (120 participants).

2. **Are any study activities ongoing outside of UHN?**

   Yes

   2a. **What study activities are ongoing outside of UHN? (Check as many as apply.):**

   Recruitment/enrolment of participants;
   Collection of data, samples, and/or study related information;
   Study interventions and/or interactions with participants;
   Participant follow-up;
   Analysis of data and/or samples;
   Transfer and/or sharing of data and/or samples

STUDY STATUS - PROSPECTIVE STUDY

What is the current status of the following study activities *at UHN*?

1.	**Recruitment/enrolment of participants**	None to date
1a.	**Specify reason why no enrollment has been done to date.** Pandemic.	
2.	**Collection of data, samples, and/or study related information**	None to date
3.	**Study interventions and/or interactions with participants**	None to date
4.	**Participant follow-up**	None to date
5.	**Analysis of data and/or samples**	None to date

6.	**Transfer and/or sharing of data and/or samples**	None to date
7.	**Manuscript preparation that requires access to participant data (including access to participants' medical records)**	None to date

CAPCR Submission Form

CAPCR-ID: 19-5857.2

Study Title: Walking while listening - the impact of hearing impairment on mobility in older adults

Study Nickname: Walking while listening

Amendment: Updated assessment list.

Date Submitted: 30 Jan 2023

PI's Name: Jennifer Campos
PI's Email: jennifer.campos@uhn.ca
PI's Phone #: 416-597-3422 Ext 7958
PI's Location: Toronto Rehabilitation Institute, University Centre, 550 University Avenue, Room 12-173, Toronto, Ontario, Canada, M5G 2A2

Study Contacts:
N/A

Prepared by:
Niroshica Mohanathas, niroshica.mohanathas@uhn.ca

Submission Documents:
Recruitment Materials (advertisement, etc.) - Appendix C. Recruitment Materials (Post)
Recruitment Materials (advertisement, etc.) - Appendix D: Recruitment Materials (Fly)
Consent Form --Tracked - Appendix G. Informed Consent Form_Tracke
Consent Form - Appendix G. Informed Consent Form_Clean
Miscellaneous Document - Appendix I. Debrief Form
Consent Form - Appendix B. Screening, Consent...
Qualitative Study Instruments (questionnaires, etc.) - Appendix E. Testing Assessments
Qualitative Study Instruments (questionnaires, etc.) - Appendix U. Subjective Cognitive
Qualitative Study Instruments (questionnaires, etc.) - Appendix V. Executive Function Training
Protocol --Tracked - Appendix A. Protocol_TRACKED
Protocol - Appendix A. Protocol_Clean
Proof of funding - Appendix B. Funding_TRACKED
Proof of funding - Appendix B. Funding_Clean
Consent-related documents (telephone script, etc.) --Tracked - Appendix E. Screening_TRACKED
Consent-related documents (telephone script, etc.) - Appendix E. Screening_Clean
Miscellaneous Document - Appendix F. Debriefing Resources_Clean
Miscellaneous Document --Tracked - Appendix F. Debriefing Resources_TRACKED
Consent Form --Tracked - Appendix G. Informed Consent_TRACKED
Consent Form - Appendix G. Informed Consent_Clean
Miscellaneous Document --Tracked - Appendix I. Debriefing_TRACKED
Miscellaneous Document - Appendix I. Debriefing_Clean

Reviewed by:
TRI KITE - StreetLab
REB (Research Ethics Board)

AMENDMENT PROFILE

**1.**	**Does this Amendment involve a change to the Principal Investigator (PI)?**	No
**2.**	**Has this amendment already been implemented to eliminate an apparent immediate hazard to one or more study participants?**	No
**3.**	**What elements of the study are affected by this Amendment? (Select all that apply.)**	
	**3.1 Study Personnel**	
	**3.1a. Study Personnel (other than PI)**	Yes
	**3.1b. Personnel who may be handling hazardous materials or transporting dangerous goods**	No
	**3.1c. UHN Health Professionals with clinical responsibilities**	No
	**3.2 Study Sites, Sponsors, and Funding**	
	**3.2a. Study sites (either within UHN or external sites)**	No
	**3.2b. Study sponsors or funding sources**	No
	**3.2c. Study budget** ***For example, due to changes in recruitment, sample size, methodology, participant compensation / reimbursement***	No
	**3.2d. Agreement or contract with any party external to UHN**	No
	**3.2e. Transfer of data, materials, human resources, etc. to/from any party outside UHN, or use of third-party software, or transfer of funds to/from UHN**	No
	**3.3 UHN facilities and services**	
	**3.3a. Services or agreements from Princess Margaret Clinical Units or CCRU Research Services**	No
	**3.3b. TRI programs, services, and facilities, including KITE Centre**	No
	**3.3c. Medical Imaging**	No
	**3.3d. Laboratory Medicine Program (LMP)**	No
	**3.3e. Health Professions (e.g. Anesthesia Assistants; Chiropody; Clinical Nutrition; Kinesiology; Lab Medicine Technicians; Nursing; Occupational Therapy; Physiotherapy; Psychology; Respiratory Therapy; Social Work; Speech Language Pathology; Spiritual Care; Therapeutic Recreation)**	No
	**3.3f. Operating Room, Anesthesia, Sterilization and/or Disinfection**	No

3.3g.	ECG	No
3.3h.	ECHO	No
3.3i.	Pulmonary Department	No
3.3j.	Apheresis	No

3.4. Study Design

3.4a.	Study Title	No
3.4b.	Study start or end date	Yes
3.4c.	Study design, including primary outcomes	Yes
3.4d.	Study populations or treatment groups/arms	Yes
3.4e.	Number of study participants	Yes
3.4f.	Number of charts to be reviewed	No
3.4g.	Number of biospecimens to be processed	No
3.4h.	Selection, monitoring, or dismissal of study participants	No
3.4i.	Evaluation of the clinical efficacy or safety of a study drug, biologic, or natural health product	No

3.5. Investigational Products

3.5a.	Study drug(s)	No
3.5b.	Natural health products or biologics	No
3.5c.	Use of investigational products, including dose, frequency of dosing, duration of treatment, manufacturing/formulation, placebo use, concommitant medications	No
3.5d.	Medical Devices	No
3.5e.	Molecular profile required for study inclusion	No

3.6. Study Data

3.6a.	Use, collection, or analysis of retrospective study data	No
3.6b.	Source(s) from which retrospective study data will be obtained	No
3.6c.	Storage of retrospective data for future use	No
3.6d.	Use, collection, or analysis of prospective study data	No
3.6e.	Collection or storage of prospective data for future use	No

**3.6f.**	**Development, purchasing, or provision of a study-specific website, application, mobile application, database, or e-tool**	No
**3.6g.**	**Use of electronic tool that collects any personal health information, transmits patient results externally, or requires a patient to enter information**	No

3.7. Biospecimens

**3.7a.**	**Addition of laboratory test(s) requiring additional samples from participants**	No
**3.7b.**	**Use, collection, or analysis of biospecimens**	No
**3.7c.**	**Source(s) from which biospecimens will be obtained**	No
**3.7d.**	**Transport or handling of biospecimens**	No
**3.7e.**	**Genetic research**	No
**3.7f.**	**Collection or storage of biospecimens for future use**	No

3.8. Risks and Safety

**3.8a.**	**Risk to health of a study participant**	No
**3.8b.**	**Use/handling of radioactive materials, including radioactive drugs**	No
**3.8c.**	**Handling or transport of hazardous materials**	No
**3.8d.**	**Use of cannabis at UHN**	No

3.9. Research Ethics

**3.9a.**	**Conflict of Interest (actual, apparent, perceived, or potential) for PI or study team member**	No
**3.9b.**	**Recruitment or consent process (e.g. how or by whom participants will be consented)**	No
**3.9c.**	**Use of personal health information**	No
**3.9d.**	**Reimbursement or compensation of study participants**	Yes
**3.9e.**	**Monitoring of study, such as DSMB or Steering Committee**	No

4. Study participants status (for UHN participants only). Check all that apply.

**4.1.**	**Study does not involve enrollment of participants (retrospective study)**	No
**4.2.**	**No enrollment to date**	No
**4.3.**	**Currently enrolling participants**	Yes

**4.4. Participants have consented but have not yet started intervention/data collection**	No
**4.5. Participants currently receiving study intervention (e.g. study drug, questionnaires, tests, or procedures done for study purposes)**	No
**4.6. Participants in post-intervention follow-up**	No
**4.7. Intervention and follow-up complete for all UHN participants** ***Data clarification, analysis, and/or transfer may be ongoing***	No
**4.8. Study involves non-UHN participants only**	No

AMENDMENT DESCRIPTION

1. **Amendment description**

   Updated assessment list.

2. **Summarize the changes to the study.**
   Where appropriate, refer to page numbers in the revised Protocol.

   Study assessments for hearing, vision and cognition have a few tests added or taken away. The user manual has been updated, a new copy

3. **Provide justification/rationale for the changes to the study.**

   Revised study documents to better align with Concordia University who will also be implementing a similar study at the PERFORM center.

4. **Has this amendment already been implemented?**

   No

5. **Will the number of participants change as a result of this amendment?**

   Yes

6. **Does this amendment require submission to Health Canada?**

   No

AMENDMENT REVIEW

1. **Are you requesting a "Full Board" REB meeting?**

   No

AMENDMENT COMMUNICATION

1. **Will study participants be informed of the changes?**

   No

   If no, complete the following question

   1b. **Explain why study participants do not need to be informed of the changes.**

   We haven't started recruiting participants yet.

Walking while listening

CAPCR ID: 19-5857.2

STUDY BASIC INFORMATION

1. **Full Study Title:**
   Walking while listening - the impact of hearing impairment on mobility in older adults
2. **Study Nickname:**
   Walking while listening
3. **Is there a protocol number or identifier for this study?**
   Yes
   If yes, complete the following question:
   3a. **Specify the protocol number/identifier.**
   19-5857.0
4. **Expected start date of the study at this institution:**
   6-Mar-2023
5. **Expected end date of the study at this institution:**
   31-Dec-2023
6. **Department/Division/Program Head:**
   Milos Popovic (milos.popovic@uhn.ca)
7. **Site(s) where this study will take place**
   TRI

STUDY OVERVIEW

1. **ABSTRACT (Suitable for a public access or lay audience):**
Navigating the environment involves successfully integrating your visual, auditory and vestibular system. However, this becomes more challenging for older adults who experience comorbid deficits in age-related hearing loss (ARHL) and mobility and are at an increased risk for falls. The "cognitive compensation" hypothesis states that hearing loss causes increased "cognitive load" because listening effort is increased. This load could take important cognitive resources away from behaviors that support safe mobility. Importantly, the interdependence of cognitive, sensory, and motor declines can lead to physical deconditioning, reduced out-of-home activity, and social withdrawal, even in individuals with sub- clinical levels of hearing loss.
2. **Background of the study: provide a summary of findings from previous studies (pre-clinical and clinical) that lead to the conduct of this study.**
   Age Effect: Although existing strategies such as hearing aids can amplify noise in quiet environments for individuals with hearing loss, these devices are limited when detecting signals in noise in more complex environments: background noise, multi-talker situations or auditory-mobility activities (e.g., balancing or walking-while-listening when crossing the street at an intersection). However, these more complex dual-tasking situations are more realistic representations of the daily situations faced by older adults. Hearing loss is also the top, mid-life potentially modifiable risk factor for dementia. This supports the idea that preventing sensory declines in middle-aged adults can be beneficial for a variety of reasons.

   Training Effect: Experimental studies provide convergent evidence of the involvement of higher-level cognitive processing in auditory task performance (e.g., speech understanding in noise, auditory working memory, language comprehension) when sensory demands are high or concurrent task demands are added. Under dual-task conditions in which a concurrent task competes for cognitive capacity, older adults exhibit greater dual-task costs in listening than younger adults, suggesting that listening becomes more cognitively effortful with aging. This recruitment of cognitive capacity to support auditory processing, even when hearing loss is sub-clinical, leaves less capacity for higher-level functions such as memory encoding, language comprehension, or other attention-demanding tasks such as walking. Given the known age-related declines in higher-level cognitive abilities such as executive function (switching,

working memory, inhibition), the potential for effective cognitive compensation could benefit from targeted Executive Function Training. Executive Function Training will especially be beneficial to those experiencing severe sensory impairments.

3. **What is the rationale for this study?**

The current study will use a state-of-the-art virtual reality simulator called Streetlab, given the complexity of sensory- motor integration required for function in the community. Both older adults and middle aged-adults will perform a battery of sensory, motor and cognitive tasks. Our main hypothesis is that all participants who undergo Executive Function Training (but not the control group) will show improved balancing and walking-while-listening task performance, with the greatest training-related gains in the ARHL group. The proposed work is novel in its inclusion of normal-hearing middle-aged adults and older hearing aid users in addition to older adults with normal hearing. This will enable us to address the timely clinical and policy-relevant issues of early prevent on and the added value of Executive Function Training in combination with hearing aid use (see Appendix B-Funding).

4. **What are the study hypotheses or research questions?**

Our main hypothesis is that all participants who undergo cognitive training (and not the waitlist control) will show improved standing, walking and listening task performance, with the greatest training-related gains in the ARHL group.

5. **Describe the primary objectives and briefly describe how they will be measured. Describe the secondary objectives and how they will be measured (if applicable).**

A) Primary Outcomes: To see the effect of cognitive training on complex auditory and motor functioning under simulated real-word conditions.

B) Secondary Outcomes: To better understand the broader impact of cognitive training, we will also assess perceptual and cognitive functioning, and subjective measures of everyday functioning before and after training.

There will be three sessions: pre-assessment, the cognitive training intervention followed by post-assessment. The pre- and post-assessment sessions include psychophysical and pencil-and-paper tasks of sensory, physical, and cognitive function. The cognitive intervention will involve training on a computer or tablet in the comfort of participant homes.

6. **What is the significance of the study (i.e. the overall anticipated public and/or scientific benefit)?**

Over 30% of individuals over age 65 experience one or more falls per year, leading to significant health care costs. The cost of fall-related injuries in Canada is estimated at $2 billion annually. Age-related mobility decline and fall risk is associated with reduced cognitive capacity, and with reduced activity of the frontal brain regions that subserve executive function and link to motor regions. The risk of falls is tripled for older adults with a hearing impairment (defined as ≥ 25 dB HL pure-tone average thresholds) compared to those with normal hearing. Yet many older adults with clinically "normal" hearing or untreated mild hearing loss experience difficulty in challenging listening environments (in background noise or multi-talker situations) or in auditory-motor activities (e.g., listening while standing, walking). A recent meta-analysis indicates that although hearing aids and auditory training can improve simple auditory task performance, Executive Function Training is needed to improve the more complex listening activities associated with everyday functioning.

MULTI-SITE STUDY

1. **What is the lead/coordinating site?**

UHN is the lead institution

2. **Indicate whether the study will be conducted at any of the TAHSN sites.**

None

3. **Who developed the protocol for this study?**

Collaborators from Concordia and UHN PI

MULTI-SITE STUDY SITES

1. **Please specify the non-UHN sites.**

   Concordia University, PERFORM Centre

STUDY PERSONNEL - PRINCIPAL INVESTIGATOR

1. **Department/Division**

   Research

2. **Program**

   N/A

3. **Site/Organization**

   TRI

   If TRI, complete the following question:

   3a. **The proposed research is associated with the PI's work as part of the following TRI research team(s):**

   Home, Community & Institutional Environments Team

4. **Does the PI require access to EPIC?**

   No

STUDY PERSONNEL

The following information is to be supplied for each member of the study team, except the PI.

Note: All personnel involved in the conduct of the study at UHN should be listed in this section, including for example, co-investigators, data abstractors and study managers. Omission of study team members from the Study Personnel list may cause a delay in the review of your submission.

**1. First and Last Name**	Niroshica Mohanathas	Lianna Montanari	Kristen Arnold
**Email**	niroshica.mohanathas@uhn.ca	lianna.montanari@uhn.ca	kristen.arnold@uhn.ca
**2. Department/Division:**	Research	N/A	N/A
**3. Program:**	Psychology	N/A	N/A
**4. Site/Organization:**	TRI	TRI	TRI
**5. Role(s) in study:**	Study Coordinator; Study Manager; Research Student	Research Student	Research Analyst
**6. Appointment expiration date (for time-limited role such as student or research fellow):**	31-Dec-2024	31-Dec-2023	31-Aug-2023
**7. Does this person require access to EPIC in order to perform this role?**	No	No	No

CONSENT

1. **Describe the consent process.**

   The consent form will be sent electronically via email to all participants after the prescreening interview (to see if participants are eligible for the study) ensuring to use the UHN file portal with the appropriate level of security for sending the document. If participants have any questions they will be encouraged to ask these questions via email or phone. Participants will sign the form when they arrive to their first study visit in the presence of the study coordinator. There will be two copies of the consent form, one for the participant to take home upon completion of the experiment and one for the research student to keep as a record.

2. **Who will obtain consent?**

   The experimenters: Niroshica Mohanathas, or other research students or research analysts.

3. **Is there a relationship between the participants and the person obtaining consent?**

   No

4. **Is there a relationship between the participants and the investigator?**

   No

5. **How much time will be given to participants to review the information before being asked to give consent?**

   As much time as they need.

6. **Does this study involve any participants who may lack the ability to provide informed consent for the duration of the study?**

   No

7. **Does this study involve any participants who may initially lack the ability to provide informed consent, but may gain or regain it at some point during the study?**

   No

8. **Does the study involve any participants who may have communication difficulties?**

   No

CONTRACTS

1. **The following information is to be supplied for each party external to UHN that will be entering into an agreement (contract) with the institution.**
   Note: A contract/agreement may be required for many reasons, for example: if the study involves multiple sites; if there is any transfer of data, materials, etc.; if there is a transfer of funds; if a medical device is being developed, built, or distributed in connection with a party outside UHN. This section is included in your CAPCR form because you have indicated that your study requires a contract or agreement. Do not answer N/A to questions in this section.

**1.1. Has the agreement been submitted (outside of CAPCR) to CTA or TDC for review and signing?**	Yes	Yes
**1.2. Has the agreement been signed by a UHN signing authority?**	Yes	Yes
**1.3. Name(s) and contact information of the other party**		

to the agreement		
1.3a. Party's full name	Karen Li	Rachel Downey
1.3b. Party's institution	Concordia	Concordia
1.3c. Party's full address	L-PY 131-4<br>Psychology Building,<br>7141 Sherbrooke W.	L-PY 131-4<br>Psychology Building,<br>7141 Sherbrooke W.
1.3d. Party's email address	Karen.Li@concordia.ca	downeyr01@gmail.com
1.4. Role of the other party:	Collaborating institution	Collaborating institution
1.5. Are biospecimens being transferred to or from this party?	No	No
1.6. Is data being transferred to or from this party?	Transfer both to and from this party	Transfer both to and from this party
1.6a. Specify what kinds of data will be transferred	Anonymous; results/data from experimental measures	Anonymous; Results/data from experimental measures.
1.7. Are funds being transferred to or from this party?	Transfer FROM this party to UHN	Transfer FROM this party to UHN

2. **Are there any other agreements or grants related to this study?**

No

DATA COLLECTION AND USE

1. **Is data being collected at UHN?**

Yes

2. **Is data analysis occurring at UHN?**

Yes

3. **Detailed description of data gathering processes and procedures**

40 middle-aged adults with normal hearing (45 - 60 years), 40 older adults with normal hearing (65-80 years) and 40 age-matched older adults (65-80 years) who are experienced hearing aid users (≥ 6 months regular use, audiometric threshold ≥ 40 dB HL @ 2 kHz) will be recruited by phone and email and will be screened over the phone and in-person for exclusion and inclusion criteria. This session has 3 parts.

The first testing session will include a battery of sensory, motor and cognitive tests. Upon completion of this session, if you are interested and eligible you will be invited for another assessment. The assessment will take place at Toronto Rehab's "virtual reality simulator. The virtual environment used in this study will resemble several city blocks surrounding Toronto Rehab. You will be asked to perform multiple trials of a) standing and walking down the street at a comfortable pace on a treadmill, b) listening to numbers and sentences and identifying numbers and words and, c) simultaneously standing and walking while performing the listening task. Upon completion of this, you will be asked to complete a series of questionnaires and computerized tasks online in the comfort of your own home for approximately 30-minute sessions, 3 times per week, for 12 weeks. After the 12 weeks you will be requested to return to Toronto Rehab to complete the sensory, motor and cognitive tests and the Virtual Reality walking-while-listening task again, which will be the third session (for additional details refer to Appendix A). Also, kinematic data (e.g., motion capture data) will be collected during the StreetLab sessions for walking.

4. **Describe the methods that will be used to analyze study data; indicate the total sample size and UHN sample size for the study. Please provide**

references to the applicable page(s) of the protocol.

We will conduct preliminary treatment group comparisons on all background variables to ensure adequate randomization across training conditions. The pre-training experimental data will be analyzed with Age Group (middle, older, ARHL) and Cognitive Load (single- vs. dual-task) as factors to confirm previously reported age and dual-task effects (e.g., auditory task accuracy, gait velocity).

Our main hypothesis is that all participants who undergo Executive Function Training (and not the active control group) will show improved walking and listening task performance, with the greatest training-related gains in the ARHL group. We will conduct mixed-factorial ANOVAs to assess the effects of Group (middle- aged, older, ARHL), Treatment (training vs. control) and Time (pre- vs. post-training) on the primary outcome variables from the StreetLab site (spatial listening accuracy, 2-back accuracy, kinematics). Should there be any group confounds detected in the preliminary analysis of the secondary outcomes (e.g., pure tone audiometry, CDTT, etc.), we will include covariates in these analyses. For the secondary outcomes, we will conduct confirmatory factor analyses to determine if the outcome measures (e.g., pure audiometry, CDTT, etc.) can be combined to form compound variables (e.g., hearing) to address the problem of multiple outcomes and spurious findings. If warranted, we will then create compound scores (averaged z-scores) and subject these to similar ANOVAs, with anticipated improvements to cognitive, motor and auditory functioning in the Executive Function Training groups. Finally, for only those participants who undergo the Executive Function Training, regression analyses will be conducted to evaluate the relationship between training gains on the Executive Function Training (mean RT slopes) and change scores on the outcome variables (refer to Appendix A for more details).

5. **Are any interim data analyses planned?**

Yes

If yes, complete the following question

5a. **Describe the interim analysis plans.**

Descriptive (e.g., mean, SD) of our dependent measures will be obtained after collecting participants data. This will be done to ensure proper data capture and integrity before collecting the full data set.

6. **Indicate how study participants will be identified in study data (e.g. study number, initials).**

Pre-screening: Initially when calling, participant's names will be linked to the pre-screening questionnaire using an ID number. The excel sheet linking participant's names and their corresponding ID number will be password protected and stored on the TRI network drive.

Recruitment: Once the participants have successfully met eligibility, their pre-screening form, consent form, health history questionnaire and all other identifying information will be de-identified and placed in a separate binder behind closed doors that require security access, separate from all testing documents.

Data Transcription and Presentation: All participants will be de-identified by using just their ID number to identify them (no identifiable information like name or email will be linked to their data from the study). All public dissemination of results will not identify any individuals and the project data will be stored for 10 years.

7. **Will any information collected through this study be linked with any other databases external to UHN? (e.g. other health care institutions, health registries, Statistics Canada)?**

No

FUNDING

1. **The following information is to be supplied for each funding source:**

**1.1. Name of company, granting agency, internal funding source, or other funding source:**	CIHR
**1.2. Type of funding source:**	Government Funding Agency - Canada (e.g. Canadian Institutes for Health Research)
**1.3. What is the status of funding from this source?**	Obtained

2. **If any requested funding is not received, will you be able to proceed with the study?**

   No

3. **If all requested funding is received, will it be sufficient to cover all study costs?**

   Yes

4. **Is this study receiving any Tri Council funding (CIHR, NSERC, SSHRC)?**

   Yes

5. **Is this study receiving any NCIC funding?**

   No

6. **Is this study receiving any US federal funds?**

   No

7. **Is this research supported by the United States federal government?**

   No

8. **Will the study require a grant account, FC (Functional Centre) or IO (Internal Order), now or in the future?**

   Yes

   If yes, complete the following question:

   8a. **Will an existing RFS account be used?**

   410012572

FUNDING - EXISTING GRANT ACCOUNT

1. **The following information is to be supplied for each existing grant account for this study:**

**1. Grant account / FC (Functional Centre) / IO (Internal Order) number from existing grant(s):**	410012572

PERSONAL HEALTH INFORMATION

1. **Specify all personal health information required to be collected for the conduct of the study, including study recruitment activities**

   Name;
   Phone/fax number;
   Email/IP address/URL;
   Date of birth (year, month, day);
   Health information (e.g. related to inclusion/exclusion criteria, medications, laboratory results)

2. **Identify sources of personal health information.**

   Directly from the participant

3. **Explain why this study cannot reasonably be accomplished without using the PHI outlined in your response above.**

   Participant's contact information (e.g., email and phone number) is used to contact the participant. In order to characterize individuals, age is required to separate participants into three groups: normal-hearing middle-aged adults (45-60 years of age) and older hearing aid users (65-80 years of age) in addition to normal- hearing older adults (65-80 years of age). In order to have the most up-to-date age we would like to collect their month, day, year to ensure their current date is reflected in analyses and publications with age (e.g., if they were tested in 2023, but the paper is published in 2024 we will know whether they are one year older depending on month/day of birth). The health and demographic information can help us ensure that all participants do indeed meet all inclusion and exclusion criteria, if something was inaccurately reported at the prescreening interview stage.

4. **Will any personal health information will be sent outside of UHN?**

   No

5. **What are the risks if PHI collected for the purposes of this study were released to an unauthorized party?**

   No Greater Than Minimal Risk: Privacy breached.

6. **If PHI were disclosed to an unauthorized party, what specific procedures, methods or controls would be implemented to minimize the potential harms?**

   Any sensitive information will be de-identified. Names will not be attached with sensitive information or asked in sensitive documents. As well, the following will be done; further release of information will be stopped, any information that can be retrieved will be retrieved, the UHN Privacy Office and REB will be notified, and then further actions may be taken according to recommendations from the UHN Privacy Office and REB.

PROSPECTIVE STUDY DESIGN

1. **Type of study**

   Lab-based behavioral study

2. **Describe the study design and methodology (provide a standalone synopsis of the study. Include: type of study (pilot, phase I, II, III, IV, RCT, qualitative, etc.), procedures (screening, intervention arm, control arm, Questionnaire, Group Discussion, Interview, etc.), duration and study visits, sub-studies (mandatory, optional, etc.)**

   We will collect information on health and demographics, eligible participants will proceed to the in-person assessment sessions.

   A) Session 1 (Pre-Training): Core Assessment (2hr):
   All participants will undergo assessment pre-training to test the following outcomes (tests also used for screening are underlined): (see Appendix A and J-M)
   (1) audition: hearing acuity pure tone audiometry and the Canadian Digit Triplets Test (CDTT)
   (2) balance and mobility: Mini-BEST
   (3) vision: ETDRS eye chart; Pelli-Robson Contrast Sensitivity Test

(4) cognition: Montreal Cognitive Assessment (MoCA), Stroop, Wechsler Adult Intelligence Scale (WAIS)-IV Digit Symbol Substitution and Letter-Number-Sequencing, Trail Making Test A&B, and Rey Auditory Verbal Learning test (RAVLT).

B) Session 1: (Pre-training) StreetLab Auditory-motor Dual-task Assessment (2 h):
We will use StreetLab to simulate everyday standing and walking (street crossing) while listening. Participants will be presented with 12 randomly ordered single digits and instructed to indicate (yes/no) if they hear a match between the current item and the one presented two items back (n-back 2). Next, participants will perform the multi-talker spatial listening task, in which a visual cue indicates which of three simultaneously presented but spatially distributed sentences to report Coordinate Response Measure (CRM).

C) Session 2: At Home Cognitive Training Intervention:
All Executive Function training participants will undergo training over 12 weeks (3x per week, 30 mins/session) on a home computer or tablet and control participants will engage in internet and computer lessons for the 12-week period.

D) Session 3: (Post-training) Core Assessments and Streetlab (2 hr)
All participants will repeat section A-B (excluding pure tone audiometry, ETDRS, Mini-BEST).

3. **Does this study include control group(s)?**

Yes

If yes, complete the following question

3a. **Indicate the rationale for control group(s).**

If the experimental group improves after training in comparison to the active control group, then the active control group is used to validate the effectiveness of training. Previous studies have shown that this active control protocol does not lead to improvements in cognition or mobility. The active control participants will get access to the cognitive training after the study is completed.

4. **Will a placebo be used?**

No

5. **Does study involve deception or intentional lack of disclosure?**

No

6. **Does this study involve qualitative components?**

Yes

If Yes, complete the following question

6a. **Specify the qualitative components of the study:**

Questionnaires/surveys

7. **Will study participants be subject to restrictions (e.g. lifestyle) during the study?**

Yes

If yes, complete the following question

7a. **Describe the restrictions for study participants.**

We ask participants not to enroll in any new exercise programs during the study.

8. **Describe the circumstances under which a participant may be withdrawn from the study.**

Participants may be withdrawn from the study if they do not meet inclusion or exclusion criteria:

Inclusion:
-Proficient in English (learned before age 5).
-Can ambulate ≥ 10 meters independently.
-Absence of cognitive impairment (MoCA score ≥ 26/30).

-Normal or corrected-to-normal visual acuity (ETDRS).
-Availability of a home computer or tablet with internet connection.
-Age (middle-aged adults: 45-60, older adults: 65-80 years of age)

Exclusions:
-Reported major depression, substance abuse or significant psychiatric disorder.
-Uncorrected visual impairment.
-Uncorrected vestibular impairment.
-Parkinson's disease or other neurological disorder or sequelae.
-Clinically significant musculoskeletal disorders, diseases affecting the ear, or damage to the ear (e.g., occupational noise).
-Onset of hearing loss prior to adulthood.

Participants have the ability to withdraw from the study at any time if they wish. Researchers will withdraw a participant from the study if they cannot complete tasks or later on meet an exclusion criteria that was not found at the prescreening interview stage of the project. However, it will be made explicitly clear in the prescreening session and/or when they come into participate through the consent form that there will be no penalties for withdrawing from the study at any time and that all data will be deleted.

PROSPECTIVE STUDY DESIGN - PARTICIPANTS

1. **Total global study enrollment (including UHN and non-UHN)**

   120

2. **Approximate size of eligible population from institution/practice (number, or number/year)**

   0

3. **Total number of participants you wish to recruit at UHN:**

   3a. **Total number of patients you wish to recruit at UHN.**

   0

   3b. **Rate of accrual (patients/month)**

   0

   3c. **Total number of non-patient participants you wish to recruit at UHN.**

   120

4. **Provide a brief summary of sample size justification. Also provide protocol reference (max 5000 characters).**

   We will recruit 3 groups:
   1) 40 middle-aged adults with normal hearing (45- 60 years old)
   2) 40 older adults with normal hearing (65+ years old)
   3) 40 age-matched older adults (65+ years old) who are experienced hearing aid users (≥ 6 months regular use, audiometric threshold ≥ 40 dB HL @ 2
   kHz).

   A convenience sample size of 40 participants in each group was chosen based on previous studies in the literature. 20 of these participants will be in the EF training group and the other 20 will be in the control group; 10 of whom are male and 10 of whom are female (refer to protocol, figure 1 for details).

5. **Time period for enrolment**

   4 months

6. **List the main inclusion and exclusion criteria pertinent to this study (max 10000 characters).**

   Inclusion:
   -Proficient in English (learned before age 5).

-Can ambulate ≥ 10 meters independently.
-Absence of cognitive impairment (MoCA score ≥ 26/30). 24 or 25 is acceptable, if they performed above cut-offs on other cognitive assessments.
-Normal or corrected-to-normal visual acuity (ETDRS).
-Availability of a home computer or tablet with internet connection.
-Age (middle-aged adults: 45-60, older adults: 65-80 years of age)

Exclusions:
-Reported major depression, substance abuse or significant psychiatric disorder.
-Uncorrected visual impairment.
-Uncorrected vestibular impairment.
-Parkinson's disease or other neurological disorder or sequelae.
-Clinically significant musculoskeletal disorders, diseases affecting the ear, or damage to the ear (e.g., occupational noise).
-Onset of hearing loss prior to adulthood.

7. **Are there any age, ethnicity, language, gender or race-related inclusion or exclusion criteria?**

Yes

If yes, complete the following question

7a. **Provide justification for inclusion/exclusion criteria.**

1) Age: We are interested in the sensory, cognitive and mobility differences between middle aged- adults and older adults with and without hearing loss. Aside from testing older adults, which has been done in the literature before, the novelty of this project is the added middle-aged group, as hearing loss is a preventable risk factor for dementia during mid-life.

2) Language: Inability to speak and understand English is required to complete tasks.

3) Gender: There is a greater prevalence and severity of hearing loss in men compared to women. Specifically, estrogen appears to have a protective effect in pre-menopausal women (e.g., sex effect) and at the same time, hearing impairment is more prevalent in those exposed to high-intensity noise which may occur more frequently in male-dominated jobs such as construction or factory work (e.g., gender effect). This is why each age group will be stratified by sex and gender.

8. **Does this study involve any of the following special populations:**

Healthy volunteers;
Staff;
Older adults, middle aged-adults, hearing loss

PUBLICATION

1. **How will results be communicated to participants?**

Individual debriefing at end of test session

2. **How will results be communicated to other stakeholders?**

Presentation;
Publication

2.1. **How will you publish the results? (Check all that apply.)**

Jointly with co-author(s) from other sites (UHN as a co-lead author with other site)

3. **Has the funding agency or sponsoring company placed any restrictions on publication of findings?**

No

RECRUITMENT - NON-PATIENTS

1. **What tools will be used to identify potential participants for recruitment into this study?**

   A list of participant's contact information who have agreed to be contacted for future studies located on our network drive and password protected; handing out and posting advertisements upon approval (e.g., posters, flyers at local areas around UHN such as, UofT, Starbucks, Tim Hortons, etc.), web-based recruitment tools [e.g., lab website (mive.ca), the Principal Investigator's (Dr. Jennifer Campos) twitter account (@jlcampos11), the research coordinator's (Niroshica Mohanathas) twitter account (@niro_mohann)].

2. **Who will identify potential study participants?**

   Graduate student (Niroshica Mohanathas) or other research students or analysts on the project.

3. **Who will make initial contact with potential participants?**

   Graduate student (Niroshica Mohanathas) or other research students or analysts on the project.

4. **How will contact be made?**

   By phone; By e-mail.

REIMBURSEMENT AND COMPENSATION

1. **Will participants be reimbursed for expenses they incur as a result of study participation?**

   No expenses will be incurred

2. **Will participants or substitute decision makers receive compensation for study participation?**

   Yes

   If yes, complete the following question

   2a. **Describe compensation (value, type), including justification.**

   You will be reimbursed $120 at the conclusion of the final session. If participants withdraw from the study due to a personal choice or due to the experimenter, they will be paid for the duration of time they completed.

3. **Who will cover reasonable out-of pocket expenses to ensure that immediate medical care is provided if a participant suffers an injury as a result of participation in this study?**

   Institution

RELATED STUDY

These questions refer to a study(ies) at this institution which is directly related to this study (e.g. sub-study, extension, rollover, subsequent to a pilot study).

**1. CAPCR ID of related study**	11-033
**2. Provide a brief summary of safety and efficacy from the related study as it relates to this submission.**	Title: Listening while Walking.<br><br>Relationship to this submission: The project builds on previous work showing that "listening is negatively affected by concurrent balance or walking demands, and that ARHL

	exacerbates dual-task costs in complex listening"; rationale for evaluating cognitive training as a potential intervention to improve functioning is developed with appropriate evidence. The current proposed study is using the n-back task and CRM task, to examine if cognitive training will improve auditory-motor performance.

RISKS, BENEFITS, SAFETY

1. **Potential benefit to study participants**

   There is no direct benefit for study participants.

2. **List the known risks of study intervention(s) in order of approximate rates of occurrence, severity and rates of reversibility (highest to lowest).**

   Overall: Risk is no greater than minimal

   Risks (highest to lowest):
   -Harness In the event of loss of balance
   -Fatigue from having multiple experimental sessions that may be considered long in duration (2 hours)
   -Discomfort from standing/sitting
   -Discomfort from eyestrain
   -Evaluation-related discomfort (from the presence of the experimenter or research assistant)

3. **List the risks of any tests, procedures or other protocol-mandated activities that are conducted for research purposes only, including approximate rates of occurrence, severity and reversibility.**

   N/A

4. **Are there any reproductive risks for participants (such as teratogenicity or embryotoxicity of the investigational product, any risk with breastfeeding, or risk to men regarding conception)?**

   No

5. **Is there a safety monitoring plan for this study?**

   Yes

   If yes, complete the following question

   5a. **Provide details of safety monitoring plan.**

   All participants will be monitored during all sessions of the experiment by the graduate student (Niroshica Mohanathas) or research students or analysts.

TRI KITE - STREETLAB

1. **Indicate the resources the study will use from this program or facility.**

   Staff;
   Equipment;
   Facilities;
   Research Data

UHN DIGITAL - SYSTEMS

1. **Please indicate what system is being used to collect personal health information; and/or transmit patient results directly to an external site/sponsor, or requires a patient to personally enter information into an electronic system (e.g. electronic survey).**

   These questionnaires will be implemented and administered using the Qualtrics online survey tool (Qualtrics, Provo, UT). Qualtrics is commonly used in psychological research. The account used in this study is a UofT Qualtrics account and has thus been approved by the UofT and the Psychology Department. All responses are completely anonymous and do not collect any identifying information. Participants will be assigned a subject code upon arrival, and this will be inputted into each survey.

A. Study Classification

1.1. Will the study at UHN be using an external ethics review process and/or system (e.g. CTO, OCREB) to obtain REB approval? No
Note: if your study is being reviewed by Veritas, please answer NO

1.2 This submission includes a proposal to: (Check all that apply.)
.

1.2a. **Investigate a current research question** Yes

1.2b. **Collect and store data and/or biospecimens for future use** No
This includes creation of a data base or biobank.

1.2c. **Extract and store retrospective data for future use** No

1.2d. **Conduct quality assurance and quality improvement studies, program evaluation activities, and performance reviews, or testing within normal educational requirements when used exclusively for assessment, management or improvement purposes** No

1.3. Is the focus of the research primarily in the field of health professions education? No

2. Does the study involve any interaction with participants? Yes
Answer YES if you are using any data that does not yet exist. For example: interventional studies; questionnaire/survey/interview; prospective (future) collection of biospecimens.
Answer NO if you are using ONLY data or samples that already exist. For example: retrospective chart review; use of previously collected biospecimens.

Does the study involve:

2.1. **Patients receiving care at Princess Margaret Cancer Centre?** No
Select yes if the intention of the study is to recruit patients

with a diagnosis of cancer or the study's objective is to prevent or screen for cancer, even if recruitment/care is occurring at TGH/TWH/TRI.

2.2. Patients receiving care at any of the Toronto Rehabilitation Institute (TRI) sites? No
This includes Toronto Rehab patients and former patients, as well as patients followed up from acute care to Toronto Rehabilitation Institute.

2.3. Patients receiving care at Toronto General Hospital? No

2.4. Patients receiving care at Toronto Western Hospital? No

2.5. Patients receiving care at any other UHN sites? No

2.6. Patients receiving care at non-UHN site(s)? No

2.7. Non-patient participants? Yes
For example: Healthy volunteers, students, health care professionals, UHN personnel

2.8. Is this a clinical trial as per the World Health Organization (WHO) definition? No

2.9. Does this study involve the use of drugs, natural health products, biologics, genetic therapies, or radioactive drugs? No

3. Does the study involve access to or use of data that does not yet exist (prospective data)? Yes
For example: medical records not yet collected, medical images/reports for scans not yet performed, questionnaires that will be collected as part of the study and databases where the data has not yet been collected.

4. Does the study involve access to or use of data that already exists (retrospective data)? No
For example: medical records that have already been collected, medical images/reports for scans that have already been performed, questionnaires that have previously been collected, databases where the data has already been collected.

5. Does the study involve use of biospecimens or their derivatives that have not yet been collected (prospective biospecimens)? No
For example: tissue, blood, body fluids, DNA, RNA, proteins, etc. that will be collected during the study

6. Does the study involve use of biospecimens or their derivatives that have already been collected (retrospective biospecimens)? No
For example: tissue, blood, body fluids, DNA, RNA, proteins, etc. that have previously been collected.

7. Are you seeking consent from any individuals for participation in this study *(UHN personnel seeking consent or seeking consent at a UHN site from any individuals for participation in this study)*? Yes

**From whom are you seeking consent?**		
**7.1.**	**Patients**<br>***For example: current or former in-patients or out-patients (UHN or non-UHN)***	No
**7.2.**	**Non-patients**<br>***For example: Healthy volunteers, students, health care professionals, UHN personnel***	Yes
**In what form will consent be obtained? (Select all that apply.)**		
**7.3.**	**Written consent with signature**	Yes
**7.4.**	**Other (e.g. verbal, implied)**	No
**7.5.**	**Do you also plan to enroll any individuals in the study without first obtaining consent?**	No
**7.6.**	**Will any personal health information be collected, used or disclosed without consent from the individuals to whom the data and/or biospecimens relate?**	No
**8.**	**Is anyone at UHN, other than the Principal Investigator, involved in the conduct of this study?**<br>***Include all personnel involved in the study, for example, staff listed on the protocol; students working for the PI; co-investigators; data abstractor; study manager, etc.***	Yes
**8a.**	**Is this research primarily conducted by personnel who are on a time-limited work term?**<br>***For example, students, fellows, residents, visiting collaborators***	Yes
**9.**	**Does this study involve research of a seasonal nature?**	No
**10.**	**Is the focus of the research primarily in the field of rehabilitation?**	Yes
**11.**	**Will any research be conducted using any staff, resources, equipment, or facilities, or any administrative, research, or health data, of the Toronto Rehabilitation Institute, including the KITE Centre for Rehabilitation Research?**	Yes
**12.**	**Is this study directly related to a previously approved study at this institution**<br>**(e.g. sub-study, extension, rollover, subsequent to a pilot study)?**	Yes
**13.**	**Is the research being conducted *exclusively* in a country other than Canada?**	No
**14.**	**Is the UHN PI the sponsor of the study (investigator-initiated study)?**	Yes
**15.**	**Does the PI have an appointment in the UHN Oncology Program?**	No

B. Risks and Safety		
**1.**	**Has this study undergone prior scientific/scholarly review?**	Yes
**2.**	**Has this study or a related study undergone review by a Research Ethics Board in Canada?**	Yes
**3.**	**Will the study have a Data and Safety Monitoring Board (DSMB)?**	No
**4.**	**Will the study have a Steering Committee?**	No
**5.**	**Will the research involve the use of Biospecimens or any other biological agent, etc. on UHN Research premises as outlined in the UHN Research Biosafety Manual?** ***Biospecimens can be blood, tissues/biopsies, primary cells, and/or body fluids (e.g.urine, stool, swab, ascites).***	No
**6.**	**Does this study involve radioactive material or radiation treatment devices? (PET scans, Bone scans, HDR, Accelerator, Brachytherapy seeds, Gammaknife)**	No
**7.**	**Will anyone participating in the study be: handling/manipulating/analysing biospecimens, and/or storing biospecimens, and/or packaging, transporting, offering for shipment, or receiving dangerous goods such as biohazardous materials, dry ice (other than radioactive materials, TDG Class 7)?**	No
**8.**	**Will any member of the study team be involved in activities with dangerous goods (other than radioactive materials, TDG Class 7) as defined by the Transportation of Dangerous Goods Regulations (TDGR)?** ***Includes packaging, transporting, offering for shipment, receiving dangerous goods such as biohazardous materials, hazardous chemicals, dry ice.***	No
**9.**	**Does anyone involved in the conduct of this study have a Conflict of Interest (actual, apparent, perceived, or potential)?**	No
**10.**	**Will the proposed research involve the use of cannabis at UHN?** ***Cannabis means a cannabis plant that belongs to the genus Cannabis and anything referred to in Schedule 1 of the Cannabis Act but does not include anything referred to in Schedule 2 of the Cannabis Act. For more information contact cannabis@uhnresearch.ca .***	No
**11.**	**Will the *storage* of study records require a deviation from the requirements of the Storage, Transport & Destruction of Confidential Information policy?**	No
**12.**	**Will the *transport* of study records require a deviation from the requirements of the Storage, Transport & Destruction of Confidential Information policy?**	No
**13.**	**Will the *destruction* of study records require a deviation from the requirements of the Storage, Transport & Destruction of Confidential Information policy?**	No

C. Resources and Services		
1.	**Does the study require any services from the Laboratory Medicine Program (LMP) at UHN?**<br>***Answer 'Yes' if any of the following is involved in the study: Anatomic Pathology; Cytopathology; Molecular/Genetic; Biochemistry; Hematopathology; Flow Cytometry; Hematology; Blood Transfusion Services (Blood Bank); Histocompatibility (HLA); Specimen Management; Microbiology; Coagulation.***	No
2.	**Does this study involve genetic research?**	No
3.	**Does the study use any medical imaging?**	No
4.	**Does the study impact ECG?**	No
5.	**Does the study impact ECHO?**	No
6.	**Is this a multicentre study (i.e. are non-UHN sites involved)?**<br>***Answer YES if any research-related activities are occurring at non-UHN sites (such as participant recruitment, enrolment, consenting, data collection, data analysis, etc.)***	Yes
7.	**Will any party external to UHN be entering into an agreement or contract with UHN in connection with this research?**	Yes
8.	**Does the study involve any transfer of data, materials, human resources, etc. to/from any party outside UHN, or use of third-party software?**<br>**"*Materials*" *includes investigational products, biological specimens, etc.***	Yes
9.	**Is there any transfer of funds to or from UHN for any purpose related to this study?**	Yes
10.	**Does the study involve any of the Health Professions, as an investigator, study participant, or carrying out study activities?**<br>***Answer YES if any of the following is involved in the study: Anesthesia Assistants; Chiropody; Clinical Nutrition; Kinesiology; Nursing; Occupational Therapy; Physiotherapy; Psychology; Radiation Therapists; Respiratory Therapy; Social Work; Speech Language Pathology; Spiritual Care; Therapeutic Recreation***	No
11.	**Do you require new space for this study?**	No
12.	**Use of Medical Devices:**<br>**Please check all that apply.**	
	**12.1. Use of software that does not have a direct impact on the diagnosis, treatment, or management of an individual's disease, disorder, abnormal physical state or symptoms**<br>***Examples:***<br>***• A tablet that is used to record information about a patient and/or symptoms***<br>***• Software intended for maintaining or encouraging a healthy lifestyle, such as general wellness apps***<br>***• Software intended to serve as electronic patient records or tools to***	No

	allow a patient to access their personal health information	
12.2.	**Developing software that is or may be a medical device** ***Refer to Health Canada Guidance Document Software as a Medical Device (SaMD): Definition and Classification and Health Canada Guidance Document Software as a Medical Device (SaMD): Classification Examples***	No
12.3.	**Use of any medical device as per the definition of "medical device" in the Canada Food and Drug Act and the Canadian Medical Devices Regulations** ***This can include hardware, software as a medical device, mobile apps, accessories, or disposables.***	No
12.4.	**Developing a medical device as per the definition of "medical device" in the Canada Food and Drug Act and the Canadian Medical Devices Regulations**	No
12.5.	**Use of medical electrical equipment that comes in physical contact with patients/participants or is used within the patient environment (within approx. 1.5 m of patient)**	No
12.6.	**No medical devices will be used in this study**	Yes
13.	**Does the study involve any reusable devices or equipment that require sterilization, high-level disinfection, or reprocessing of equipment or medical devices?**	No
14.	**Does the study involve the use of the Operating Room?**	No
15.	**Does the study require or alter provision of care by the Anesthesia Care Team (ACT)?**<br>***The Anesthesia Care Team includes: Anesthesiology staff and trainees, Anesthesia Assistants, and Pain Specialists including Pain Nurse Practitioners.***<br>***Scope of care of ACT includes:***<br>***1) all procedures requiring sedation/anesthesia in the operating room, cath lab, JDMI, endoscopy, POCU and PACU.***<br>***2) acute, transitional, and chronic pain medicine at TGH/TWH/PMH.***<br>***3) provision of care in the CVICU***<br>***4) provision of care in the hyperbaric medicine unit and***<br>***5) conduct of trans-esophageal echocardiography in the operating room and CVICU.***	No
16.	**Does the study impact the Pulmonary Department?**	No
17.	**Does the study impact Apheresis?**	No
18.	**Does this study require the services of the Cancer Clinical Research Unit (CCRU)?**	No
19.	**Does the study require the development, purchasing, or provision of a study specific website, application, mobile application, database, or e-tool (e.g. IOS or Android app, new REDCap project, etc.)?**	No
20.	**Does the study require the use of an electronic tool that collects any**	Yes

personal health information; and/or transmits patient results directly to an external site/sponsor, or requires a patient to personally enter information into an electronic system (e.g. electronic survey)?

Indicate the system(s) used:

**20a.**	**Existing web applications hosted and maintained by UHN (e.g. CRR, Medidata, etc.)**	No
**20b.**	**A sponsor-provided eCRF system, application, or medical device that directly transmits patient results (e.g. sponsor provided ECG machine that sends results directly to the sponsor)**	No
**20c.**	**An externally sourced website or application that is NOT hosted by UHN Digital, that patients enter information into (e.g. to complete surveys or self-assessments)**	Yes
**20d.**	**Other**	No

**21.**	**Does this study require the use of UHN Biospecimen Services?**	No

G. TRI Facilities

Which of the following TRI programs and/or facilities will be used in connection with this study?

**1.**	**Brain** ***Including Acquired Brain Injury (ABI), Stroke, LIFEspan, Chronic Pain, Multiple Sclerosis (MS), Complex Injury Outpatient Rehabilitation (CIOR), and Neurology***	No
**2.**	**Spinal Cord**	No
**3.**	**Cardiac Rehab**	No
**4.**	**Geriatric Rehab**	No
**5.**	**Geriatric Psychiatry**	No
**6.**	**Musculoskeletal**	No
**7.**	**Ambulatory Care**	No
**8.**	**South 5 - Complex Continuing Care**	No
**9.**	**South 4 - Low Tolerance Long Duration (LTLD)**	No
**10.**	**South 3 - Complex Continuing Care including Dialysis**	No
**11.**	**North 3 - Transitional Care Unit (TCU)**	No
**12.**	**North 5 - Complex and Continuing Care (CCC)**	No
**13.**	**North 5 - Transitional Care Unit (TCU)**	No
**14.**	**Speciality Clinics and Augmentative and Alternative Communication (AAC)**	No

	**Clinic**	
**15.**	**University Center (UC) Dental Services**	No
**16.**	**Bickle Center (BC) Dental Services**	No
**17.**	**KITE HomeLab**	No
**18.**	**KITE CareLab**	No
**19.**	**KITE FallsLab**	No
**20.**	**KITE ClimateLab**	No
**21.**	**KITE WinterLab**	No
**22.**	**KITE StreetLab**	Yes
**23.**	**KITE StairLab**	No
**24.**	**KITE DriverLab**	No
**25.**	**KITE PerceptionLab**	No
**26.**	**KITE RampLab**	No
**27.**	**SwallowingLab**	No
**28.**	**SleepdBLab**	No
**29.**	**Sleep Clinic**	No
**30.**	**TACOSLab**	No
**31.**	**Movement EvaluationLab**	No
**32.**	**Mobility InnovationsCentre**	No
**33.**	**RELLab**	No
**34.**	**Rumsey CardiacLab**	No
**35.**	**Rumsey NeuroLab**	No
**36.**	**Rocket Family Upper Extremity Clinic-UC**	No
**37.**	**Rocket Family Upper Extremity Clinic-LC**	No
**38.**	**SCI MobilityLab**	No
**39.**	**Other TRI program/facilities**<br>***Additional information must be supplied in the TRI NOTES section***	No

CAPCR Submission Form

CAPCR-ID: 19-5857.3

Study Title: Walking while listening - the impact of hearing impairment on mobility in older adults

Study Nickname: Walking while listening

Research Ethics Renewal

Date Submitted: 22 Sep 2022

PI's Name: Jennifer Campos
PI's Email: jennifer.campos@uhn.ca
PI's Phone #: 416-597-3422 Ext 7958
PI's Location: Toronto Rehabilitation Institute, University Centre, 550 University Avenue, Room 12-173, Toronto, Ontario, Canada, M5G 2A2

Study Contacts:
N/A

Prepared by:
Niroshica Mohanathas, niroshica.mohanathas@uhn.ca

Submission Documents:
N/A

Reviewed by:
REB (Research Ethics Board)

Expiry Date: 28-Sep-2025

Full Board Meeting requested: No

Walking while listening

CAPCR ID: 19-5857.3

STUDY SUMMARY - PROSPECTIVE STUDY

1. **Provide a brief summary of the progress of the study to date (e.g. recruitment issues, preliminary findings, qualitative study enrolment).**
   For multicentre studies: specify total sample size and number of participants enrolled at other centers.
   If the study is conducted in phases, e.g. dose escalation and dose expansion parts; Phase I and Phase II; indicate which Part/Phase is currently enrolling participants.

   Haven't started collecting data due to the pandemic.

2. **Is there any new information in the literature or from other recent studies that would change the rationale or risk/benefit ratio for this study (e.g. changes in standard of care, new information about side effects, approval of another drug for this indication, etc.)?**

   No

3. **Have any participants been withdrawn from the study intervention prematurely, or withdrawn consent?**

   No

4. **Have there been any participant complaints or feedback about the study?**

   No

5. **Since the last renewal, have all reportable events (e.g. unexpected deaths or serious adverse events related to study participation, etc.) been reviewed in a timely fashion by the PI?**

   There have not been any reportable events since the last renewal

6. **Since the last renewal, have all reportable events been reported to the REB?**

   There have not been any reportable events since the last renewal

7. **Is the study being conducted in accordance with the documents currently approved by the UHN Research Ethics Board?**

   Yes

8. **Since the last renewal, has there been any change in the Conflict of Interest information for study personnel involved in this study, that has not been reported to the REB?**

   No

9. **Are you requesting a "Full Board" REB meeting?**

   No

**10.**	**Is this study receiving any US federal funds?**	No
**11.**	**Is this research supported by the United States federal government?**	No

STUDY PARTICIPANTS - PROSPECTIVE STUDY (RENEWAL)

1.	**Total number of participants approved by the UHN REB to be enrolled at UHN**	120
2.	**Number of charts reviewed to determine eligibility for enrollment**	0
3.	**Number of participants consented to date at UHN**	0
3a.	**Number of patient participants**	0
3b.	**Number of non-patient participants**	0
4.	**Number of Participants:**	
4.1.	**Consented but did not meet inclusion criteria**	0

4.2.	**Consented but have not yet started intervention/data collection**	0
4.3.	**Receiving study intervention (e.g. study drug, questionnaires, tests, or procedures done for study purposes)**	0
4.4.	**In post-intervention follow-up**	0
4.5.	**Have completed the study and no further contact for study purposes is planned**	0
4.6.	**Have withdrawn their consent**	0
4.7.	**Have been withdrawn prematurely by the PI**	0

STUDY PARTICIPANTS - MULTICENTRE STUDY

1. **For multicentre studies, indicate the number of participants enrolled at other centres.**

   To be determined- data collection has been slow due to the pandemic. However, this external institution is aiming to collect the same sample size as our UHN site (120 participants).

2. **Are any study activities ongoing outside of UHN?**

   Yes

   2a. **What study activities are ongoing outside of UHN? (Check as many as apply.):**

   Recruitment/enrolment of participants;
   Collection of data, samples, and/or study related information;
   Study interventions and/or interactions with participants;
   Participant follow-up;
   Analysis of data and/or samples;
   Transfer and/or sharing of data and/or samples

STUDY STATUS - PROSPECTIVE STUDY

What is the current status of the following study activities *at UHN*?

1.	**Recruitment/enrolment of participants**	None to date
1a.	**Specify reason why no enrollment has been done to date.** Pandemic.	
2.	**Collection of data, samples, and/or study related information**	None to date
3.	**Study interventions and/or interactions with participants**	None to date
4.	**Participant follow-up**	None to date
5.	**Analysis of data and/or samples**	None to date
6.	**Transfer and/or sharing of data and/or samples**	None to date
7.	**Manuscript preparation that requires access to participant data (including access to participants' medical records)**	None to date

CAPCR Submission Form

CAPCR-ID: 19-5857.4

Study Title: Walking while listening - the impact of hearing impairment on mobility in older adults

Study Nickname: Walking while listening

Research Ethics Renewal

Date Submitted: 26 Sep 2023

PI's Name: Jennifer Campos
PI's Email: jennifer.campos@uhn.ca
PI's Phone #: 416-597-3422 Ext 7958
PI's Location: Toronto Rehabilitation Institute, University Centre, 550 University Avenue, Room 12-173, Toronto, Ontario, Canada, M5G 2A2

Study Contacts:
N/A

Prepared by:
Niroshica Mohanathas, niroshica.mohanathas@uhn.ca

Submission Documents:
N/A

Reviewed by:
REB (Research Ethics Board)

Expiry Date: 28-Sep-2025

Full Board Meeting requested: No

Walking while listening

CAPCR ID: 19-5857.4

STUDY SUMMARY - PROSPECTIVE STUDY		
1.	**Provide a brief summary of the progress of the study to date (e.g. recruitment issues, preliminary findings, qualitative study enrolment).<br>For multicentre studies: specify total sample size and number of participants enrolled at other centers.<br>If the study is conducted in phases, e.g. dose escalation and dose expansion parts; Phase I and Phase II; indicate which Part/Phase is currently enrolling participants.**<br>Data collection is ongoing.	
2.	**Is there any new information in the literature or from other recent studies that would change the rationale or risk/benefit ratio for this study (e.g. changes in standard of care, new information about side effects, approval of another drug for this indication, etc.)?**<br>No	
3.	**Have any participants been withdrawn from the study intervention prematurely, or withdrawn consent?**<br>No	
4.	**Have there been any participant complaints or feedback about the study?**<br>No	
5.	**Since the last renewal, have all reportable events (e.g. unexpected deaths or serious adverse events related to study participation, etc.) been reviewed in a timely fashion by the PI?**<br>There have not been any reportable events since the last renewal	
6.	**Since the last renewal, have all reportable events been reported to the REB?**<br>There have not been any reportable events since the last renewal	
7.	**Is the study being conducted in accordance with the documents currently approved by the UHN Research Ethics Board?**<br>Yes	
8.	**Since the last renewal, has there been any change in the Conflict of Interest information for study personnel involved in this study, that has not been reported to the REB?**<br>No	
9.	**Are you requesting a "Full Board" REB meeting?**<br>No	
**10.**	**Is this study receiving any US federal funds?**	No
**11.**	**Is this research supported by the United States federal government?**	No

STUDY PARTICIPANTS - PROSPECTIVE STUDY (RENEWAL)		
1.	**Total number of participants approved by the UHN REB to be enrolled at UHN**	120
2.	**Number of charts reviewed to determine eligibility for enrollment**	0
3.	**Number of participants consented to date at UHN**	0
	3a. **Number of patient participants**	0
	3b. **Number of non-patient participants**	0
4.	**Number of Participants:**	
	4.1. **Consented but did not meet inclusion criteria**	0

4.2.	Consented but have not yet started intervention/data collection	0
4.3.	Receiving study intervention (e.g. study drug, questionnaires, tests, or procedures done for study purposes)	0
4.4.	In post-intervention follow-up	0
4.5.	Have completed the study and no further contact for study purposes is planned	0
4.6.	Have withdrawn their consent	0
4.7.	Have been withdrawn prematurely by the PI	0

STUDY PARTICIPANTS - MULTICENTRE STUDY

1. **For multicentre studies, indicate the number of participants enrolled at other centres.**

   Concordia University has recruited 23 middle-aged adults, 29 older adults with normal-hearing and 11 older adult hearing aid users. They have completed data collection and are in the process of looking at the data now.

2. **Are any study activities ongoing outside of UHN?**

   Yes

   2a. **What study activities are ongoing outside of UHN? (Check as many as apply.):**

   Analysis of data and/or samples;
   Transfer and/or sharing of data and/or samples

STUDY STATUS - PROSPECTIVE STUDY

What is the current status of the following study activities *at UHN*?

1.	Recruitment/enrolment of participants	Ongoing
2.	Collection of data, samples, and/or study related information	Ongoing
3.	Study interventions and/or interactions with participants	Ongoing
4.	Participant follow-up	Ongoing
5.	Analysis of data and/or samples	Ongoing
6.	Transfer and/or sharing of data and/or samples	Ongoing
7.	Manuscript preparation that requires access to participant data (including access to participants' medical records)	Ongoing

CAPCR Submission Form

CAPCR-ID: 19-5857.5

Study Title: Walking while listening - the impact of hearing impairment on mobility in older adults

Study Nickname: Walking while listening

Research Ethics Renewal

Date Submitted: 12 Sep 2024

PI's Name: Jennifer Campos
PI's Email: jennifer.campos@uhn.ca
PI's Phone #: 416-597-3422 Ext 7958
PI's Location: Toronto Rehabilitation Institute, University Centre, 550 University Avenue, Room 12-173, Toronto, Ontario, Canada, M5G 2A2

Study Contacts:
N/A

Prepared by:
Niroshica Mohanathas, niroshica.mohanathas@uhn.ca

Submission Documents:
N/A

Reviewed by:
REB (Research Ethics Board)

Expiry Date: 28-Sep-2025

Full Board Meeting requested: No

STUDY SUMMARY - PROSPECTIVE STUDY

1. **Provide a brief summary of the progress of the study to date (e.g. recruitment issues, preliminary findings, qualitative study enrolment).**
   For multicentre studies: specify total sample size and number of participants enrolled at other centers.
   If the study is conducted in phases, e.g. dose escalation and dose expansion parts; Phase I and Phase II; indicate which Part/Phase is currently enrolling participants.

   Data collection is completed and we are now analyzing and writing up the data.

2. **Is there any new information in the literature or from other recent studies that would change the rationale or risk/benefit ratio for this study (e.g. changes in standard of care, new information about side effects, approval of another drug for this indication, etc.)?**

   No

3. **Have any participants been withdrawn from the study intervention prematurely, or withdrawn consent?**

   Yes

   3a. Provide the reasons for participant withdrawal.

   Can't participate in the study due to illness, change in travel plans or time commitment.

4. **Have there been any participant complaints or feedback about the study?**

   No

5. **Since the last renewal, have all reportable events (e.g. unexpected deaths or serious adverse events related to study participation, etc.) been reviewed in a timely fashion by the PI?**

   There have not been any reportable events since the last renewal

6. **Since the last renewal, have all reportable events been reported to the REB?**

   There have not been any reportable events since the last renewal

7. **Is the study being conducted in accordance with the documents currently approved by the UHN Research Ethics Board?**

   Yes

8. **Since the last renewal, has there been any change in the Conflict of Interest information for study personnel involved in this study, that has not been reported to the REB?**

   No

9. **Are you requesting a "Full Board" REB meeting?**

   No

10. **Is this study receiving any US federal funds?** No

11. **Is this research supported by the United States federal government?** No

Walking while listening

CAPCR ID: 19-5857.5

STUDY PARTICIPANTS - PROSPECTIVE STUDY (RENEWAL)

1.	**Total number of participants approved by the UHN REB to be enrolled at UHN**	120
2.	**Number of charts reviewed to determine eligibility for enrollment**	0
3.	**Number of participants consented to date at UHN**	0
3a.	**Number of patient participants**	0
3b.	**Number of non-patient participants**	0
4.	**Number of Participants:**	
4.1.	**Consented but did not meet inclusion criteria**	0
4.2.	**Consented but have not yet started intervention/data collection**	0
4.3.	**Receiving study intervention (e.g. study drug, questionnaires, tests, or procedures done for study purposes)**	0
4.4.	**In post-intervention follow-up**	0
4.5.	**Have completed the study and no further contact for study purposes is planned**	0
4.6.	**Have withdrawn their consent**	0
4.7.	**Have been withdrawn prematurely by the PI**	0

STUDY PARTICIPANTS - MULTICENTRE STUDY

1. **For multicentre studies, indicate the number of participants enrolled at other centres.**

   Concordia University has recruited 23 middle-aged adults, 29 older adults with normal-hearing and 11 older adult hearing aid users. They have completed data collection and are in the process of looking at the data now.

2. **Are any study activities ongoing outside of UHN?**

   Yes

   2a. **What study activities are ongoing outside of UHN? (Check as many as apply.):**

   Analysis of data and/or samples;
   Transfer and/or sharing of data and/or samples

STUDY STATUS - PROSPECTIVE STUDY

What is the current status of the following study activities *at UHN*?

1.	**Recruitment/enrolment of participants**	Complete
2.	**Collection of data, samples, and/or study related information**	Complete
3.	**Study interventions and/or interactions with participants**	Complete
4.	**Participant follow-up**	Complete
5.	**Analysis of data and/or samples**	Ongoing
6.	**Transfer and/or sharing of data and/or samples**	Ongoing
7.	**Manuscript preparation that requires access to participant data (including access to participants' medical records)**	Ongoing
