# Supplementary material for: Effects of executive function training on balance and auditory-cognitive dual-task performance in adults with and without hearing loss
Source: PLoS One. 2026 Apr 29;21(4):e0331276. doi: 10.1371/journal.pone.0331276 (PMC13127936; doi:10.1371/journal.pone.0331276)
Supplement: S4 File — Study overview. (DOCX) [file pone.0331276.s006.docx]

**Study Overview**

**Brief Summary**

The proposed study is designed to evaluate the effect of at-home executive function training on cognition and mobility in older adults with age-related hearing loss (ARHL), older adults with normal hearing, and middle-aged adults.

**Official Title**

Training Cognition to Improve Mobility and Listening in Older Adults with Hearing Loss: Moving from Lab to Life

**Conditions**

Aging Age Related Hearing Loss

**Intervention / Treatment**

- Behavioral: Executive function (EF) training

**Other Study ID Numbers**

- 30011799

**Study Start (Actual)**

2020-08-30

**Primary Completion (Actual)**

2023-08-15

**Study Completion (Actual)**

2024-08-15

**Enrollment (Actual)**

63

**Study Type**

Interventional

**Phase**

Not Applicable

**Resource links provided by the National Library of Medicine**

[MedlinePlus Genetics](https://medlineplus.gov/genetics/) related topics:  [Age-related hearing loss](https://medlineplus.gov/genetics/condition/age-related-hearing-loss)

[FDA Drug and Device Resources](https://clinicaltrials.gov/fda-links)

**Contacts and Locations**

This section provides contact details for people who can answer questions about joining this study, and information on where this study is taking place.

To learn more, please see the [Contacts and Locations section in How to Read a Study Record](https://clinicaltrials.gov/study-basics/how-to-read-study-record#contacts-and-locations).

This study has 1 location

**Canada**

**Quebec Locations**

**[Montreal, Quebec, Canada, H3G 1M8](https://clinicaltrials.gov/)**

[Concordia University](https://clinicaltrials.gov/)

**Participation Criteria**

Researchers look for people who fit a certain description, called eligibility criteria. Some examples of these criteria are a person's general health condition or prior treatments.

For general information about clinical research, read [Learn About Studies](https://clinicaltrials.gov/study-basics/learn-about-studies).

**Eligibility Criteria**

**Description**

Inclusion Criteria:

- Proficient in English (learned before age 5), can ambulate ≥ 10 meters independently, absence of cognitive impairment (neuropsychological test results in the average range compared to age norms), normal or corrected-to-normal visual acuity (ETDRS), availability of a home computer or tablet with internet connection.

Exclusion Criteria:

- Reported major depression, substance abuse or significant psychiatric disorder, uncorrected visual impairment, vestibular impairment, Parkinson's disease or other neurological disorder or sequelae, clinically significant musculoskeletal disorders, diseases affecting the ear, or damage to the ear (e.g., occupational noise), onset of hearing loss prior to adulthood.

**Ages Eligible for Study**

45 Years to 80 Years (Adult,  Older Adult )

**Sexes Eligible for Study**

All

**Accepts Healthy Volunteers**

Yes

**Study Plan**

This section provides details of the study plan, including how the study is designed and what the study is measuring.

Expand all / Collapse all

How is the study designed?

**Design Details**

**Primary Purpose  :**Prevention

**Allocation  :**Randomized

**Interventional Model  :**Parallel Assignment

**Masking  :**Single (Investigator)

**Masking Description:**The investigator will be blinded to group assignment.

**Arms and Interventions**

| **Participant Group/Arm** | **Intervention/Treatment** |
| --- | --- |
| Experimental: Executive function (EF) training  12 weeks of at-home EF training on a computer or iPad | Behavioral: Executive function (EF) training   - EF training will involve at-home computer- or tablet based training to improve several EF processes, including divided attention, response inhibition, switching, and working memory updating. Training session duration = 30 min x 3 session/week. |
| No Intervention: Wait-list control  This will be a comparator arm with no cognitive training. Participants will be given access to the same training program following the conclusion of the study, but no further assessment is planned. |  |

| **Outcome Measure** | **Measure Description** | **Time Frame** |
| --- | --- | --- |
| Pre-training Change in auditory 2-back accuracy | Auditory 2-back performance will be assessed under single- and dual-task conditions (i.e., while the participant is standing or simultaneously walking on a treadmill at a self-selected pace). The numbers for the task will be played through binaural speakers, and the decibel level will be individualized for each participant depending on hearing ability. Participants will be required to make a button response indicating whether the number they heard was the same or different from the number they heard two previously. Accuracy (percent correct) will be recorded. Half the participants will be given this assessment. See Outcome 5, Multi-talker, for other half. | baseline |
| Post-training Change in auditory 2-back accuracy | Auditory 2-back performance will be assessed under single- and dual-task conditions (i.e., while the participant is standing or simultaneously walking on a treadmill at a self-selected pace). The numbers for the task will be played through binaural speakers, and the decibel level will be individualized for each participant depending on hearing ability. Participants will be required to make a button response indicating whether the number they heard was the same or different from the number they heard two previously. Accuracy (percent correct) will be recorded. Half the participants will be given this assessment. See Outcome 6, Multi-talker, for other half. | after 12 weeks |
| Pre-training Change in auditory 2-back reaction time | Auditory 2-back performance will be assessed under single- and dual-task conditions (i.e., while the participant is standing or simultaneously walking on a treadmill at a self-selected pace). The numbers for the task will be played through binaural speakers, and the decibel level will be individualized for each participant depending on hearing ability. Participants will be required to make a button response indicating whether the number they heard was the same or different from the number they heard two previously. Reaction time (msec) will be recorded. Half the participants will be given this assessment. See Outcome 5, Multi-talker, for other half. | baseline |
| Post-training Change in auditory 2-back reaction time | Auditory 2-back performance will be assessed under single- and dual-task conditions (i.e., while the participant is standing or simultaneously walking on a treadmill at a self-selected pace). The numbers for the task will be played through binaural speakers, and the decibel level will be individualized for each participant depending on hearing ability. Participants will be required to make a button response indicating whether the number they heard was the same or different from the number they heard two previously. Reaction time (msec) will be recorded. Half the participants will be given this assessment. See Outcome 6, Multi-talker, for other half. | after 12 weeks |
| Pre-training Multi-Talker word perception (accuracy) | We will use the multi-talker spatial listening task for the other half of the participants, in which a presented text cue indicates which of three simultaneously presented, but spatially distributed spoken sentences to report. Traffic noise will be included to more closely simulate real- world conditions. The signal-to-noise ratio will be held constant across participants, with signal intensity set according to individual average hearing thresholds. | baseline |
| Post-training Multi-Talker word perception (accuracy) | We will use the multi-talker spatial listening task for the other half of the participants, in which a presented text cue indicates which of three simultaneously presented, but spatially distributed spoken sentences to report. Traffic noise will be included to more closely simulate real- world conditions. The signal-to-noise ratio will be held constant across participants, with signal intensity set according to individual average hearing thresholds. | after 12 weeks |
| Pre-training Change in stride time (mean, standard deviation [SD] of stride time) | Stride time will be assessed under single- and dual-task conditions (i.e., walking on a treadmill at a self-selected pace or while simultaneously completing the auditory 2-back task). The spatio-temporal characteristics of gait will be measured by foot pressure sensors worn on the base of the participants' shoes, with one sensor placed beneath the heel and the second sensor placed beneath the toe. The time between successive toe or heel strikes will be recorded (sec) and averaged across the trials. Those participants being assessed with Outcome 5 will perform the same walking task but in a simulated street-crossing environment. | baseline |
| Post-training Change in stride time (mean, standard deviation [SD] of stride time) | Stride time will be assessed under single- and dual-task conditions (i.e., walking on a treadmill at a self-selected pace or while simultaneously completing the auditory 2-back task). The spatio-temporal characteristics of gait will be measured by foot pressure sensors worn on the base of the participants' shoes, with one sensor placed beneath the heel and the second sensor placed beneath the toe. The time between successive toe or heel strikes will be recorded (sec) and averaged across the trials. Those participants being assessed with Outcome 6 will perform the same walking task but in a simulated street-crossing environment. | after 12 weeks |
| Pre-training Change in stride time variability (mean, standard deviation [SD] of stride time) | Stride time variability will be assessed under single- and dual-task conditions (i.e., walking on a treadmill at a self-selected pace or while simultaneously completing the auditory 2-back task). The spatio-temporal characteristics of gait will be measured by foot pressure sensors worn on the base of the participants' shoes, with one sensor placed beneath the heel and the second sensor placed beneath the toe. The time between successive toe or heel strikes will be recorded (sec) and the standard deviation will be taken to derive a measure of stride time variability. Those participants being assessed with Outcome 5 will perform the same walking task but in a simulated street-crossing environment. | baseline |
| Post-training Change in stride time variability (mean, standard deviation [SD] of stride time) | Stride time variability will be assessed under single- and dual-task conditions (i.e., walking on a treadmill at a self-selected pace or while simultaneously completing the auditory 2-back task). The spatio-temporal characteristics of gait will be measured by foot pressure sensors worn on the base of the participants' shoes, with one sensor placed beneath the heel and the second sensor placed beneath the toe. The time between successive toe or heel strikes will be recorded (sec) and the standard deviation will be taken to derive a measure of stride time variability. Those participants being assessed with Outcome 6 will perform the same walking task but in a simulated street-crossing environment. | after 12 weeks |

| **Outcome Measure** | **Measure Description** | **Time Frame** |
| --- | --- | --- |
| Pre-training Montreal Cognitive Assessment (MoCA) | Neuropsychological test to evaluate global cognitive status. Scored on 30, a score of 26 or higher is used to identify Mild Cognitive Impairment (MCI) however a score of 23 or higher is used in this study as this cut-off has been shown to lower false positivity. (Nasreddine et al., 2005) | baseline |
| Post-training Montreal Cognitive Assessment (MoCA) | Neuropsychological test to evaluate global cognitive status. Scored on 30, a score of 26 or higher is used to identify Mild Cognitive Impairment (MCI) however a score of 23 or higher is used in this study as this cut-off has been shown to lower false positivity. (Nasreddine et al., 2005) | after 12 weeks |
| Pre-training WAIS-IV Digit Symbol Coding | Neuropsychological test to evaluate processing speed. Subtest of the Wechsler Adult Intelligence Scale IV (WAIS; Wechsler, 2008). Scored based on correct number of symbols completed in 120 seconds. | baseline |
| Post-training WAIS-IV Digit Symbol Coding | Neuropsychological test to evaluate processing speed. Subtest of the Wechsler Adult Intelligence Scale IV (WAIS; Wechsler, 2008). Scored based on correct number of symbols completed in 120 seconds. | after 12 weeks |
| Pre-training Trail Making Test | Neuropsychological test to evaluate processing speed and executive functioning (Reitan, 1992). In form A, participants use a pencil to connect circles on a page in ascending numerical order. In form B, participants connect circles, alternating between ascending number and letter series. Time to complete (s) is recorded per form. Difference between Forms B and A completion times provides an index of switching, updating, and is considered a measure of executive function. | baseline |
| Post-training Trail Making Test | Neuropsychological test to evaluate processing speed and executive functioning (Reitan, 1992). In form A, participants use a pencil to connect circles on a page in ascending numerical order. In form B, participants connect circles, alternating between ascending number and letter series. Time to complete (s) is recorded per form. Difference between Forms B and A completion times provides an index of switching, updating, and is considered a measure of executive function. | after 12 weeks |
| Pre-training D-KEFS Stroop | Delis-Kaplan Executive Function System (D-KEFS) Color-Word Interference Test (CWIT) is a neuropsychological test to evaluate processing speed and executive functioning (adapted from Delis, Kaplan, & Kramer, 2001). A modification of the Stroop task (Stroop, 1935). In the CWIT, participants are asked to complete four conditions: colour naming, reading (both discontinued after 90 seconds), inhibition, and inhibition/switching (both discontinued after 180 seconds). Number correctly completed per condition. | baseline |
| Post-training D-KEFS Stroop | Delis-Kaplan Executive Function System (D-KEFS) Color-Word Interference Test (CWIT) is a neuropsychological test to evaluate processing speed and executive functioning (adapted from Delis, Kaplan, & Kramer, 2001). A modification of the Stroop task (Stroop, 1935). In the CWIT, participants are asked to complete four conditions: colour naming, reading (both discontinued after 90 seconds), inhibition, and inhibition/switching (both discontinued after 180 seconds). Number correctly completed per condition. | after 12 weeks |
| Pre-training Rey Auditory Verbal Learning Test | Neuropsychological test to evaluate verbal memory (RAVLT; Rey, A. (1941). Scored by: adding up to correctly recalled words for each trial to obtain immediate recall total; how many correctly recalled words after the 30-minute interval to obtain their delayed recall total; divided delayed recall total by the total words correctly recalled on the fifth (last) trial of the initial administration and multiplied by 100 to obtain their percentage retention value. | baseline |
| Post-training Rey Auditory Verbal Learning Test | Neuropsychological test to evaluate verbal memory (RAVLT; Rey, A. (1941). Scored by: adding up to correctly recalled words for each trial to obtain immediate recall total; how many correctly recalled words after the 30-minute interval to obtain their delayed recall total; divided delayed recall total by the total words correctly recalled on the fifth (last) trial of the initial administration and multiplied by 100 to obtain their percentage retention value. | after 12 weeks |
| Pre-training WAIS-IV Digit span | Neuropsychological test to evaluate short-term memory. Subtest of the Wechsler Adult Intelligence Scale IV (WAIS; Wechsler, 2008). Scored based on highest span length correctly completed. | baseline |
| Post-training WAIS-IV Digit span | Neuropsychological test to evaluate short-term memory. Subtest of the Wechsler Adult Intelligence Scale IV (WAIS; Wechsler, 2008). Scored based on highest span length correctly completed. | after 12 weeks |
| Pre-training Subjective listening self efficacy | Listening self-efficacy questionnaire (LSEQ: Smith, Pichora-Fuller, Watts, & La More, 2011, Int J Audiol). Asks questions about one's self-rated ability to understand conversation in a variety of contexts, without the use of hearing aids, self-rated self-efficacy (0-100%). Internal consistency (Chronbach's α) = .96 (Smith et al., 2011). | baseline |
| Post-training Subjective listening self efficacy | Listening self-efficacy questionnaire (LSEQ: Smith, Pichora-Fuller, Watts, & La More, 2011, Int J Audiol). Asks questions about one's self-rated ability to understand conversation in a variety of contexts, without the use of hearing aids. Internal consistency (Chronbach's α) = .96 (Smith et al., 2011). | after 12 weeks |
| Pre-training Subjective balance confidence | ABC Balance Confidence Questionnaire (Powell & Myers, 1995, J Geront: MedSci). Sixteen-item questionnaire concerning self-rated confidence (0-100%) balancing in different physical activities and contexts. | baseline |
| Post-training Subjective balance confidence | ABC Balance Confidence Questionnaire (Powell & Myers, 1995, J Geront: MedSci). Sixteen-item questionnaire concerning self-rated confidence (0-100%) balancing in different physical activities and contexts. | after 12 weeks |
| Pre-training Mobility functioning as assessed by the Mini BESTest | The Mini BESTest is comprised of four sub-scales to evaluate balance performance, including an anticipatory sub-scale (e.g., sit-to-stand, toe rise), a reactive postural control subscale (measures compensatory stepping when one's centre of balance is displaced in the forward, backward, or lateral position), a sensory orientation subscale (e.g., eyes closed, foam mat), and a gait subscale (e.g., walking over an obstacle). | baseline |
| Post-training Mobility functioning as assessed by the Mini BESTest | The Mini BESTest is comprised of four sub-scales to evaluate balance performance, including an anticipatory sub-scale (e.g., sit-to-stand, toe rise), a reactive postural control subscale (measures compensatory stepping when one's centre of balance is displaced in the forward, backward, or lateral position), a sensory orientation subscale (e.g., eyes closed, foam mat), and a gait subscale (e.g., walking over an obstacle). | after 12 weeks |
| Pre-training Functional neuroimaging (fNIRS) | Portable functional near infrared spectroscopy (BRITE MKII) will be used to assess prefrontal cortex activity during the single- and dual-task walking conditions. Sixteen detectors will be placed strategically 2.8 cm away from the optodes, eight of them will be dorsal to the optodes, while the other eight will be ventral, so that each probe has four dorsal detectors and four ventral detectors. The two probes will be placed symmetrically over the lateral prefrontal cortex and the most anterior and most ventral pair of optode-detector of each probe will be placed on Fp1/Fp2. The probes were designed based on existing configurations. Of interest are relative changes in concentration of frontal oxy-hemoglobin (HbO2: measured at absorption peak of 850nm) and deoxy-hemoglobin (HbR: measured at absorption peak of 735nm) compared to the baseline rest data. | baseline |
| Post-training Functional neuroimaging (fNIRS) | Portable functional near infrared spectroscopy (BRITE MKII) will be used to assess prefrontal cortex activity during the single- and dual-task walking conditions. Sixteen detectors will be placed strategically 2.8 cm away from the optodes, eight of them will be dorsal to the optodes, while the other eight will be ventral, so that each probe has four dorsal detectors and four ventral detectors. The two probes will be placed symmetrically over the lateral prefrontal cortex and the most anterior and most ventral pair of optode-detector of each probe will be placed on Fp1/Fp2. The probes were designed based on existing configurations. Of interest are relative changes in concentration of frontal oxy-hemoglobin (HbO2: measured at absorption peak of 850nm) and deoxy-hemoglobin (HbR: measured at absorption peak of 735nm) compared to the baseline rest data. | after 12 weeks |
| Pre-training Standing Balance (Path length) | Postural measures will include spatial measures (centre of pressure path length; cm) in the anterior-posterior (front and back) and medial-lateral (side-to-side) orientations. | baseline |
| Pre-training Standing Balance (Velocity) | Postural measures will include temporal measures (velocity; cm/s) in the anterior-posterior (front and back) and medial-lateral (side-to-side) orientations. | baseline |
| Pre-training Standing Balance (Variability) | Postural measures will include variability measures (root means square, standard deviation) in the anterior-posterior (front and back) and medial-lateral (side-to-side) orientations. | baseline |
| Post-training Standing Balance (Path length) | Postural measures will include spatial measures (centre of pressure path length; cm) in the anterior-posterior (front and back) and medial-lateral (side-to-side) orientations. | after 12 weeks |
| Post-training Standing Balance (Velocity) | Postural measures will include temporal measures (velocity; cm/s) in the anterior-posterior (front and back) and medial-lateral (side-to-side) orientations. | after 12 weeks |
| Post-training Standing Balance (Variability) | Postural measures will include variability measures (root means square, standard deviation) in the anterior-posterior (front and back) and medial-lateral (side-to-side) orientations. | after 12 weeks |

What is the study measuring?

**Primary Outcome Measures**

**Secondary Outcome Measures**

**Collaborators and Investigators**

This is where you will find people and organizations involved with this study.

**Sponsor**

**Concordia University, Montreal**

**Collaborators**

- Canadian Institutes of Health Research (CIHR)

**Publications**

**From PubMed**

These publications come from PubMed, a public database of scientific and medical articles. This list is automatically created by ClinicalTrials.gov Identifier (NCT Number), and these articles may or may not be about the study.

- [Downey R, Gagne N, Mohanathas N, Campos JL, Pichora-Fuller KM, Bherer L, Lussier M, Phillips NA, Wittich W, St-Onge N, Gagne JP, Li K. At-home computerized executive-function training to improve cognition and mobility in normal-hearing adults and older hearing aid users: a multi-centre, single-blinded randomized controlled trial. BMC Neurol. 2023 Oct 20;23(1):378. doi: 10.1186/s12883-023-03405-1.](https://pubmed.ncbi.nlm.nih.gov/37864139)

**Study Record Dates**

These dates track the progress of study record and summary results submissions to ClinicalTrials.gov. Study records and reported results are reviewed by the National Library of Medicine (NLM) to make sure they meet specific quality control standards before being posted on the public website.

**Study Registration Dates**

**First Submitted**

2022-05-26

**First Submitted that Met QC Criteria**

2022-06-09

**First Posted**

2022-06-15

**Study Record Updates**

**Last Update Submitted that met QC Criteria**

2025-03-12

**Last Update Posted**

2025-03-14

**Last Verified**

2025-03

**More Information**

Expand all / Collapse all

**Terms related to this study**

**Keywords Provided by Karen Li, Concordia University, Montreal**

Cognitive training

Executive function

Aging

Dual-task walking

Near-infrared spectroscopy

Hearing loss

Middle age

Mobility

**Additional Relevant MeSH Terms**

Hearing Loss, Sensorineural

Hearing Disorders

Ear Diseases

Otorhinolaryngologic Diseases

Sensation Disorders

Neurologic Manifestations

Nervous System Diseases

Signs and Symptoms

Pathological Conditions, Signs and Symptoms

Presbycusis

Hearing Loss

**Plan for Individual Participant Data (IPD)**

**Plan to Share Individual Participant Data (IPD)?**

Yes

**Drug and device information, study documents, and helpful links**

**Studies a U.S. FDA-Regulated Drug Product**

No

**Studies a U.S. FDA-Regulated Device Product**

No

**Product Manufactured in and Exported from the U.S.**

No
